# The Role of Sialyl Glycan Recognition in Host Tissue Tropism of the Avian Parasite *Eimeria tenella*

**DOI:** 10.1371/journal.ppat.1002296

**Published:** 2011-10-13

**Authors:** Livia Lai, Janene Bumstead, Yan Liu, James Garnett, Maria A. Campanero-Rhodes, Damer P. Blake, Angelina S. Palma, Wengang Chai, David J. P. Ferguson, Peter Simpson, Ten Feizi, Fiona M. Tomley, Stephen Matthews

**Affiliations:** 1 Division of Molecular Biosciences, Imperial College London, South Kensington Campus, London, United Kingdom; 2 Institute for Animal Health, Compton, Newbury, Berkshire, United Kingdom; 3 Glycosciences Laboratory, Department of Medicine, Imperial College London, Hammersmith Campus, London, United Kingdom; 4 Department of Pathology and Infectious Diseases, Royal Veterinary College, North Mymms, Hatfield, Hertfordshire, United Kingdom; 5 Nuffield Department of Clinical Laboratory Science, Oxford University, John Radcliffe Hospital, Oxford, United Kindgom; MRC National Institute for Medical Research, United Kingdom

## Abstract

*Eimeria* spp. are a highly successful group of intracellular protozoan parasites that develop within intestinal epithelial cells of poultry, causing coccidiosis. As a result of resistance against anticoccidial drugs and the expense of manufacturing live vaccines, it is necessary to understand the relationship between *Eimeria* and its host more deeply, with a view to developing recombinant vaccines. *Eimeria* possesses a family of microneme lectins (MICs) that contain **m**icroneme **a**dhesive **r**epeat **r**egions (MARR). We show that the major MARR protein from *Eimeria tenella*, EtMIC3, is deployed at the parasite-host interface during the early stages of invasion. EtMIC3 consists of seven tandem MAR1-type domains, which possess a high specificity for sialylated glycans as shown by cell-based assays and carbohydrate microarray analyses. The restricted tissue staining pattern observed for EtMIC3 in the chicken caecal epithelium indicates that EtMIC3 contributes to guiding the parasite to the site of invasion in the chicken gut. The microarray analyses also reveal a lack of recognition of glycan sequences terminating in the *N*-glycolyl form of sialic acid by EtMIC3. Thus the parasite is well adapted to the avian host which lacks *N*-glycolyl neuraminic acid. We provide new structural insight into the MAR1 family of domains and reveal the atomic resolution basis for the sialic acid-based carbohydrate recognition. Finally, a preliminary chicken immunization trial provides evidence that recombinant EtMIC3 protein and EtMIC3 DNA are effective vaccine candidates.

## Author Summary

*Eimeria* spp. are highly successful protozoan parasites of the intestine of birds and one of the most important diseases in modern poultry farming. The economic impact is significant causing billion dollar losses to the industry and as a result there is pressing need for new therapeutic approaches. Anticoccidial drugs are thwarted by resistance, live vaccines are expensive to manufacture and few recombinant vaccine antigens have been characterized in detail. We show that the microneme protein, MIC3 from *Eimeria tenella*, is deployed at the parasite-host interface during the early stages of invasion. We provide new atomic resolution insight into its predilection for sialic acid-bearing glycans and demonstrate its role in invasion. We also provide evidence that EtMIC3-based vaccines induce protection in preliminary immunization studies.

## Introduction

The phylum Apicomplexa contains some of the most widespread protozoan parasites of humans and animals. Key members include *Plasmodium* spp., *Eimeria* spp., *Neospora caninum* and *Toxoplasma gondii*. *Eimeria* spp. are a highly successful group of host-specific, intracellular protozoan parasites that develop within intestinal epithelial cells, causing Coccidiosis, which is economically one of the most important diseases in modern poultry farming, and causes billion dollar economic losses worldwide [1]. The importance of the poultry industry is highlighted by the effort to develop anticoccidial drugs, however many of these have been thwarted by drug resistance. The incorporation of vaccination with avirulence strains of *Eimeria* has vastly improved the control of infections. The development of recombinant vaccines is hampered by a relatively poor molecular understanding of the *Eimeria*-host interface.

Infection by apicomplexans is established in the host by rapid and forced invasion of host cells using a multistep process [2]. It begins with an initial phase of non-oriented cell attachment then a search across cellular surfaces for a particular niche and finally deployment of the cell entry machinery. Microneme proteins secreted in the early stages of this process participate in attachment to the host cell and subsequent formation of the connection with the parasite actinomyosin system, thereby providing the platform from which to drive invasion [3]. A family of microneme lectins (MICs) has been described that recognize sialylated glycans via microneme adhesive repeat regions (MARR) [4]. The first MARR protein to be characterized was MIC1 from *Toxoplasma gondii* (TgMIC1), which possesses a pair of N-terminal MARR that form two distinct subfamilies based on sequence, MAR1 and MAR2. TgMIC1 recognizes sialic acid (Sia)-terminating glycan chains: a wide variety of sialyl linkages including α2–3, α2–6 and α2–8 that are abundantly present on host cell surfaces. This broad specificity likely contributes to *T. gondii*'s ability to establish an infection in all warm-blooded animals [4], [5]. *T. gondii* also possesses additional MARR proteins that further extend the repertoire of sialylated cell-surface glycoconjugates recognized by this parasite [6]. High resolution structures have highlighted a critical TxH motif in the MAR2 domain that coordinates the sialyl moiety [4], [5]. Intriguingly, MARR are also present in proteins of enteric coccidian parasites with very specific host and tissue tropisms, such as *Eimeria spp*. that exhibit strong site-specificity of development in the chicken intestine. *Eimeria tenella* develops within cells of the caecum and caecal tonsils located at the ileocecal junction, whereas *Eimeria acervulina* infects cells of the duodenum and *Eimeria maxima* infects cells of the jejunum [7]. *E. tenella* possesses a MARR-containing microneme protein, EtMIC3, which is composed of seven tandem MARR that belong exclusively to the MAR1 family [3], [8], [9] (Figure S1).

In this paper we provide a detailed structural, biochemical and cellular characterization of EtMIC3. We demonstrate that EtMIC3 is composed of tandem MAR1 domains that possess a high specificity for sialylated glycans that terminate in *N*-acetylneuraminic acid (NeuAc), but not in *N*-glycolylneuraminic acid (NeuGc). The ability of EtMIC3 to bind to α2–3 sialyl sequences and their abundance in the chicken caecal epithelium, are likely to contribute to directing the parasite to this specific location in the chicken gut. Furthermore, we demonstrate that recombinant EtMIC3 protein or DNA act as an effective vaccine.

## Results

### Localization and host cell binding of EtMIC3

**Figure 1:**
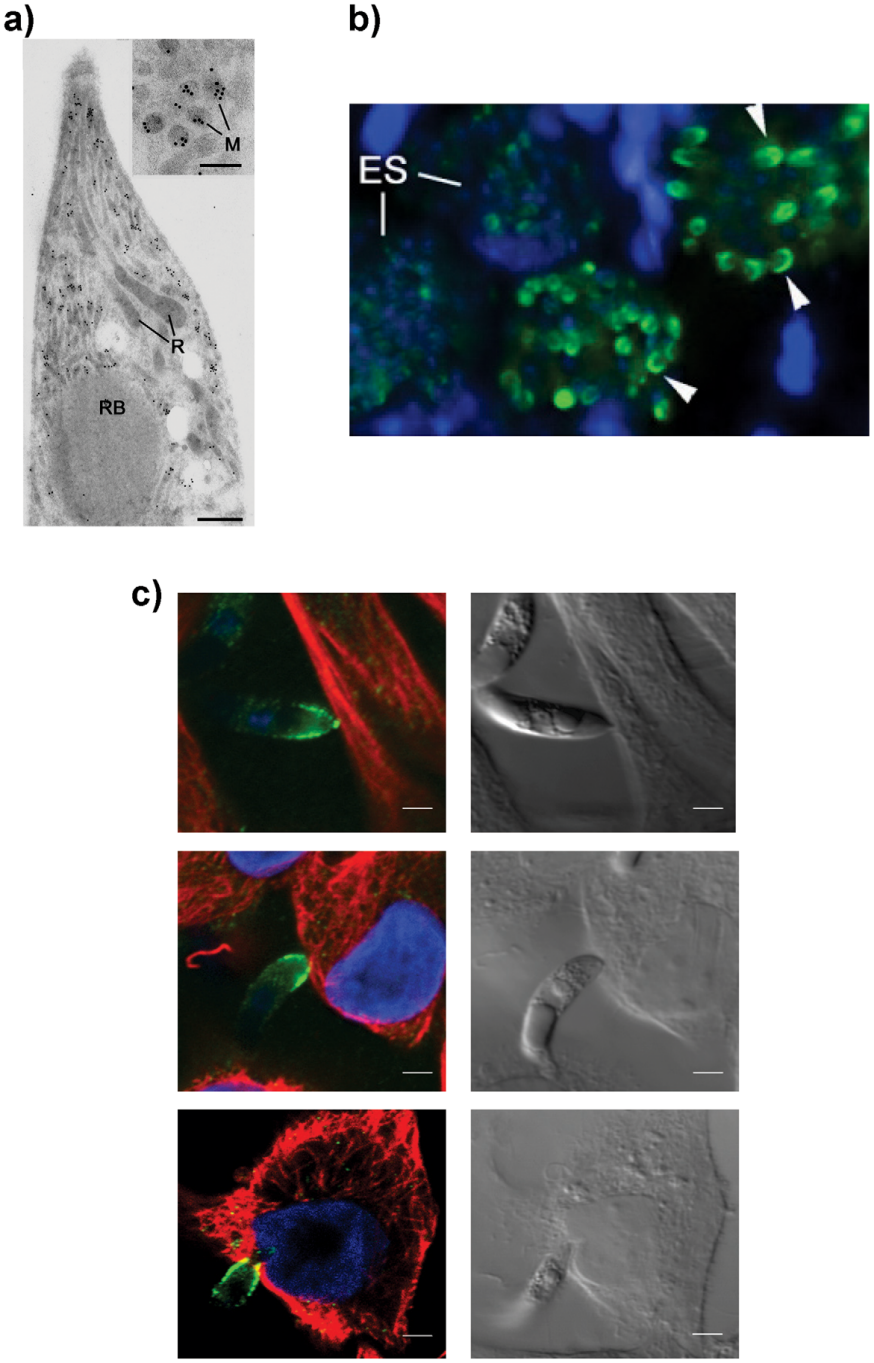
EtMIC3 is a microneme protein. (**A**) TEM/immunogold localization of EtMIC3 in an *E. tenella* sporozoite shows it to be located exclusively to the micronemes. (R) rhoptry, (M) microneme (RB) refractile body. The bar represents 1 µm and 0.1 µm in insert. (**B**) Immunofluorescence localization of EtMIC3 in fixed and permeabilized developing schizonts of *E. tenella* within a section of infected caecum shows it to be located at the apical tips of newly formed merozoites in a crescent shaped distribution. Blue counterstain is DAPI. Arrowheads indicate merozoites emerging from mature schizonts. (ES) early schizonts. The bar represents 10 µm. (**C**) Immunofluorescence localization (left panel) and corresponding differential interference contrast (DIC, right panel) of EtMIC3 (green) and beta tubulin (red) in fixed and permeabilized invading *E. tenella* sporozoites on MDBK monolayers. Blue counterstain is DAPI. Sporozoite attaches to the host cell (top panel) causing invagination of the host cell membrane (middle panel) and the sporozoite becomes committed to invasion with an extruded conoid (bottom panel). Note merged labelling of EtMIC3 and host cell tubulin at the moving junction (yellow) in the bottom panel. EtMIC3 is present at the apical end of the sporozoite throughout these early invasion stages. The bar represents 1 µm.

**Figure 2:**
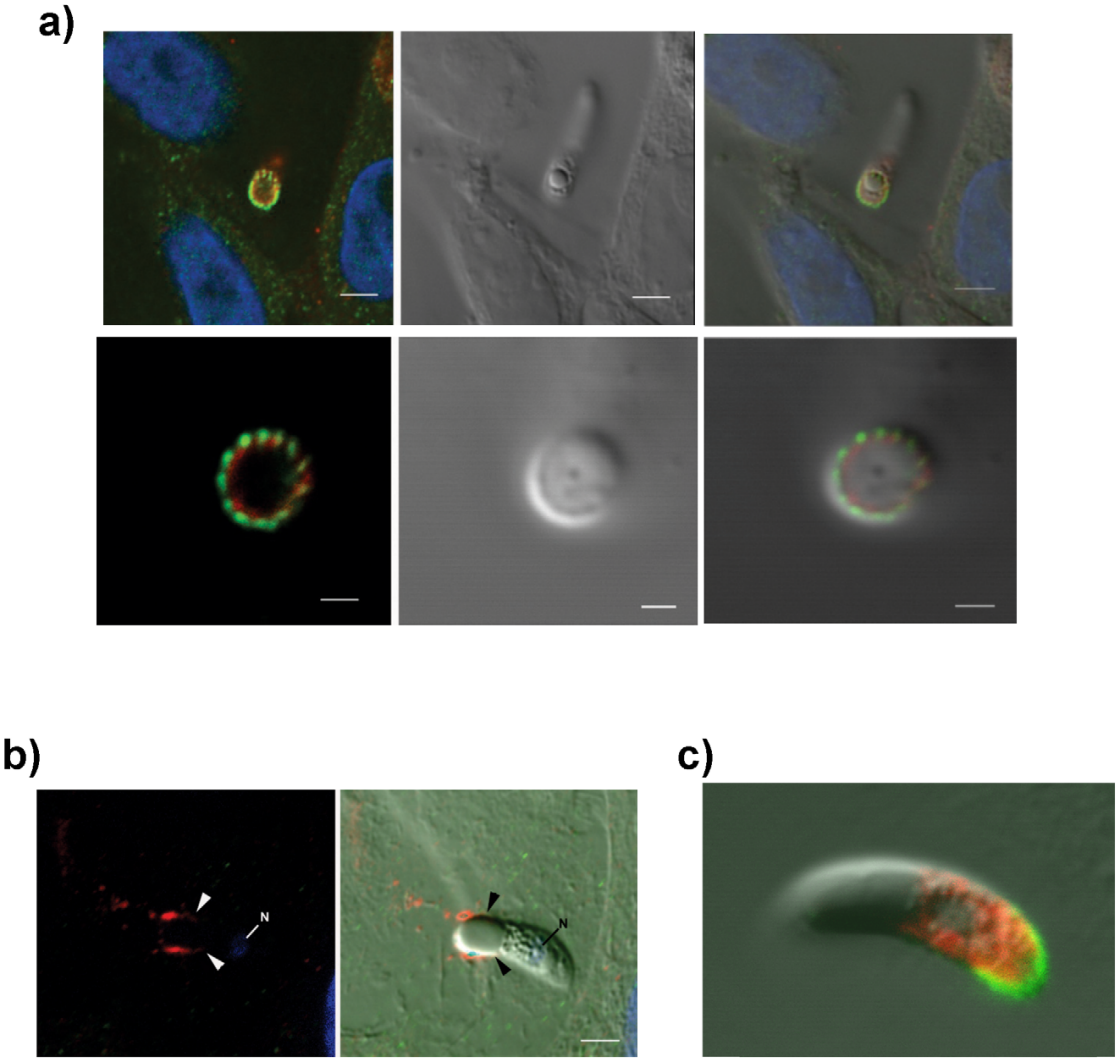
Details of localization of EtMIC3 and EtAMA1 in invading parasites. (**a**) Immunofluorescence localisation (left, top and bottom panels) and DIC (right, top and bottom panels) of EtMIC3 (green) and EtAMA1 (red) in fixed and permeabilised invading *E. tenella* sporozoites on MDBK monolayers. Blue counterstain is DAPI. Both EtMIC3 and EtAMA1 label necklace- like structures present at the junction between the invading parasite and the host cell; the labelling is closely associated but does not co-localize. The top panels indicate the position of the parasite-host junction and the bottom panels give a clear cross sectional image of the non-overlapping staining patterns. The bar represents 2 µm in top panels, 1 µm in bottom panels. (**b**) Immunofluorescence localisation (left panel) of EtMIC3 (red) and EtAMA1 (green) in fixed, unpermeabilized invading *E. tenella* sporozoite (left image) and combined with DIC (right panel). The parasite is invading from left to right and EtMIC3 is found on the surface at the region of the moving junction (arrowheads) and as a trail on the host cell surface. EtAMA1 was not detected. Blue counterstain is DAPI, N indicates parasite nucleus. The bar represents 2 µm. (**c**) Immunofluorescent localization of EtMIC3 (green) and EtMIC5 (red) in permeabilised *E. tenella* sporozoite attached to MDBK cell in culture. EtMIC3 is concentrated at the extended conoid and the apical surface of the sporozoite whereas EtMIC5 is slightly posterior to this region. The bar represents 3 µm.

To confirm the intraparasite localization for EtMIC3 prior to secretion, immunogold labeling of *E. tenella* sporozoites with anti-EtMIC3 antibodies was performed and visualized using transmission electron microscopy (TEM). As shown in Figure 1a, gold particles decorate exclusively the microneme compartments. The polar location of EtMIC3 was confirmed by immunofluorescence microscopy (IFA; Figure 1b) showing localization in merozoites developing within schizonts in the chicken caecum. To follow the localization of EtMIC3 during invasion *E. tenella* sporozoites were incubated with monolayers of Madin-Darby bovine kidney (MDBK) cells, which were then fixed, permeabilized and examined by immunofluorescence assay (IFA) and differential interference contrast (DIC) microscopy (Figure 1c). After attachment to the host cell, the sporozoite caused invagination of the host cell membrane and became committed to invasion with an extruded conoid. EtMIC3 was present at the apical surface of the sporozoite throughout these early invasion stages (Figure 1c) and during invasion was detected at the interface between the host and parasite cell membranes where it was present on a bead like structure that forms a tight ring around the apical perimeter of the sporozoite (Figure 2).

To examine more precisely the point at which EtMIC3 functions in invasion, the localization of EtMIC3 was compared to that of two other important microneme proteins EtAMA1, an integral component of the moving junction together with rhoptry neck (RON) proteins [10] and EtMIC5 [11], [12], [13], a lactose-binding, secreted microneme protein. In fixed and permeabilized parasites, EtMIC3 labeled a necklace structure comprising 14 separate foci present at the junction between the invading parasite and the host cell. This was proximal to a similar staining pattern for EtAMA1, but not aligned exactly (Figure 2a). In contrast, visualization of fixed, non-permeabilized sporozoites revealed that during invasion EtMIC3 is detected around the circumference of the parasite at the host cell-parasite junction and deposited on the host cell surface, whereas EtAMA1 is not labeled (Figure 2b). This suggests that whilst EtAMA1 is buried within the moving junction and, in the absence of permeabilization, not accessible to antibody, EtMIC3 is more peripherally associated with the junction and remains surface exposed and in contact with the host cell. Dual immunofluorescence staining of EtMIC3 and EtMIC5 was also performed on fixed and permeabilized sporozoites and in parasites that were apically attached to host cells EtMIC3 serum gave a strong signal at the apical tip of the sporozoite, whilst the majority of EtMIC5 labeling was detected just posterior to this region (Figure 2c), indicating that it was not yet secreted.

### EtMIC3 targets cell surface sialic acid-bearing molecules

**Figure 3:**
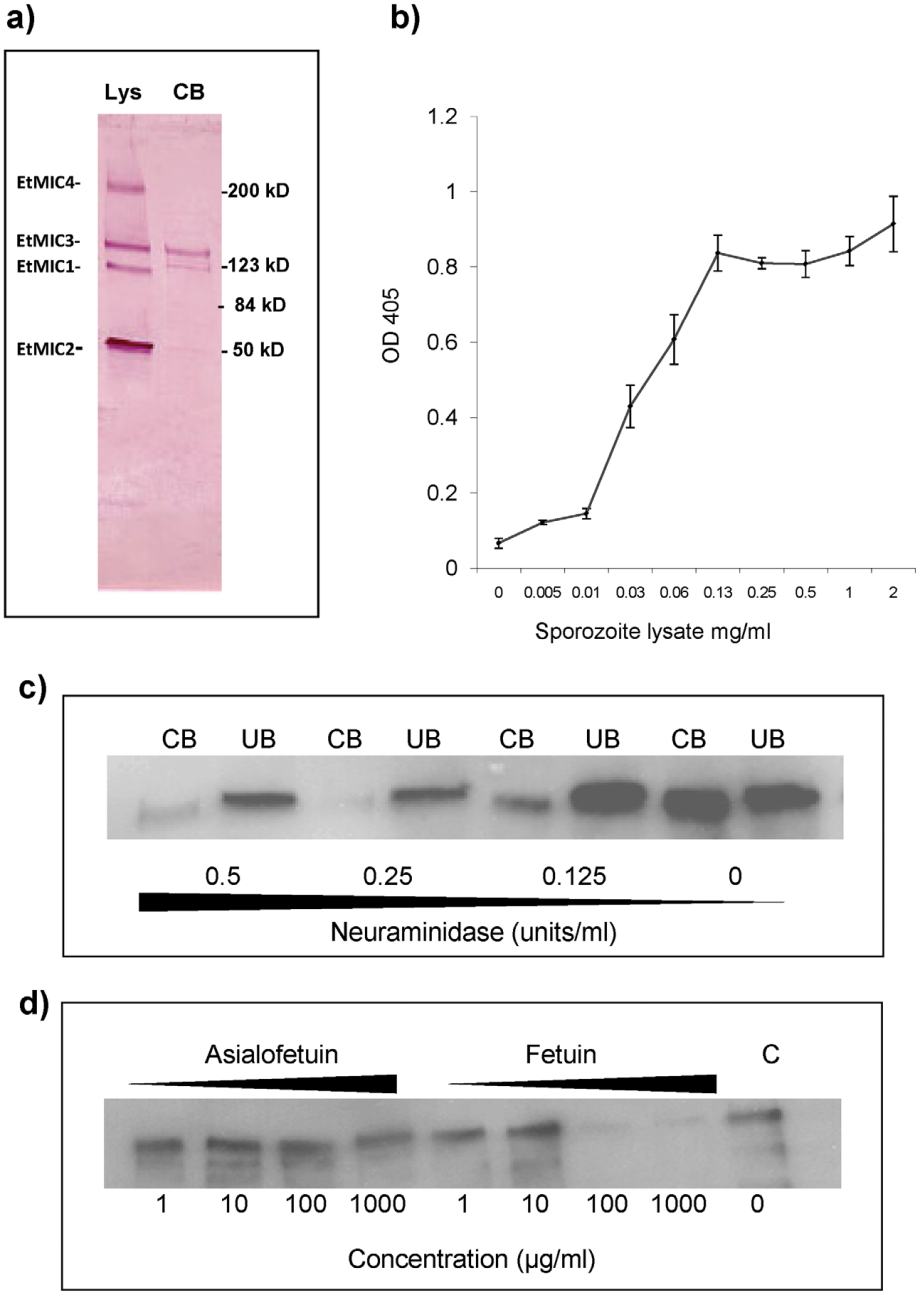
EtMIC3 binds cell surface sialylated carbohydrates. (**a**) Western blot analysis of a sporozoite lysate (lys) and the cell-bound (CB) fraction of the lysate after incubation on MDBK cells. Proteins were detected using specific antisera to EtMIC1, EtMIC2, EtMIC3 and EtMIC4. All four proteins were detected in the sporozoite lysate, but only EtMIC3 was detected in the cell bound protein fraction of this assay this. It is useful to note that cell binding activity has been reported for the EtMIC4/5 complex [11], but the assay protocol used in this work is less sensitive and unable to detect it. The faint band below EtMIC3 is likely to be a break down product. (**b**) Dose-dependent bindg of a sporozoite lysate containing EtMIC3 to fixed MDBK cell monolayers determined by cell based ELISA. Sporozoite lysate was diluted in PBS at a range of concentrations from 2 mg/ml - 0.001 mg/ml. The lysate was incubated with gluteraldehyde fixed MDBK cell monolayers. Cells were washed to remove unbound protein and binding of EtMIC3 was determined by ELISA. Error bars indicate standard deviations. (**c**) Western blot analysis of EtMIC3 from a sporozoite lysate (20×10^6^/ml) within cell bound (CB) or unbound (UB) fractions of a sporozoite lysate in the presence of neuraminidase (c), (**d**) fetuin or asialofetuin.

Sporozoite lysates were incubated with monolayers of MDBK cells and proteins that bound to the cells identified by Western blotting. Whilst EtMIC1, EtMIC2, EtMIC3 and EtMIC4 proteins were readily detected in the unbound fraction of the sporozoite lysate, only EtMIC3 was observed to any extent in the cell bound fraction (Figure 3a). Subsequent ELISA type cell-based binding assays performed with sporozoite lysate showed a dose dependent increase in the bound fraction of EtMIC3 (Figure 3b).

EtMIC3 possesses a tandem MARR, and the MARRs in the MIC proteins of *T. gondii*, and *N. caninum* have been shown to recognize a variety of sialylated glycans [4], [5], [6]. To establish whether host cell sialyl glycans are a target for EtMIC3, cell binding assays were performed in the presence of exo-α-sialidase (neuraminidase), which strips cell surface sialic acid residues, or the presence of fetuin, a sialylated glycoprotein derived from bovine fetal serum, which would compete with sialyl oligosaccharides on the cell surface for MIC3 binding. Treatment with neuraminidase at 0.25 units per ml or fetuin at 100 µg per ml effectively inhibited binding of EtMIC3 to host cells (Figure 3c & 3d). In contrast, treatment with asialofetuin, a glycoprotein that lacks sialic acid and instead has terminal galactose residues, did not compete for EtMIC3 binding. Interestingly, treatment with free sialic acid (NeuAc) did not inhibit EtMIC3 binding, which is in sharp contrast with our observation with TgMIC1 whose binding is inhibited by free sialic acid [4], [6]. Fetuin and multi-sialylated gangliosides containing at least one terminal α2–3 sialyl linkage were extremely potent inhibitors of EtMIC3 binding. The GD1a disialo-ganglioside which possesses both terminal and side chain α2–3 sialic acid moieties is a potent inhibitor of cell binding, whereas the related GD1b disialo-ganglioside, in which only the α2–8 di-sialyl side chain is present, was not (Figure S2). This is in accord with the results of microarray analyses (see below).

**Figure 4:**
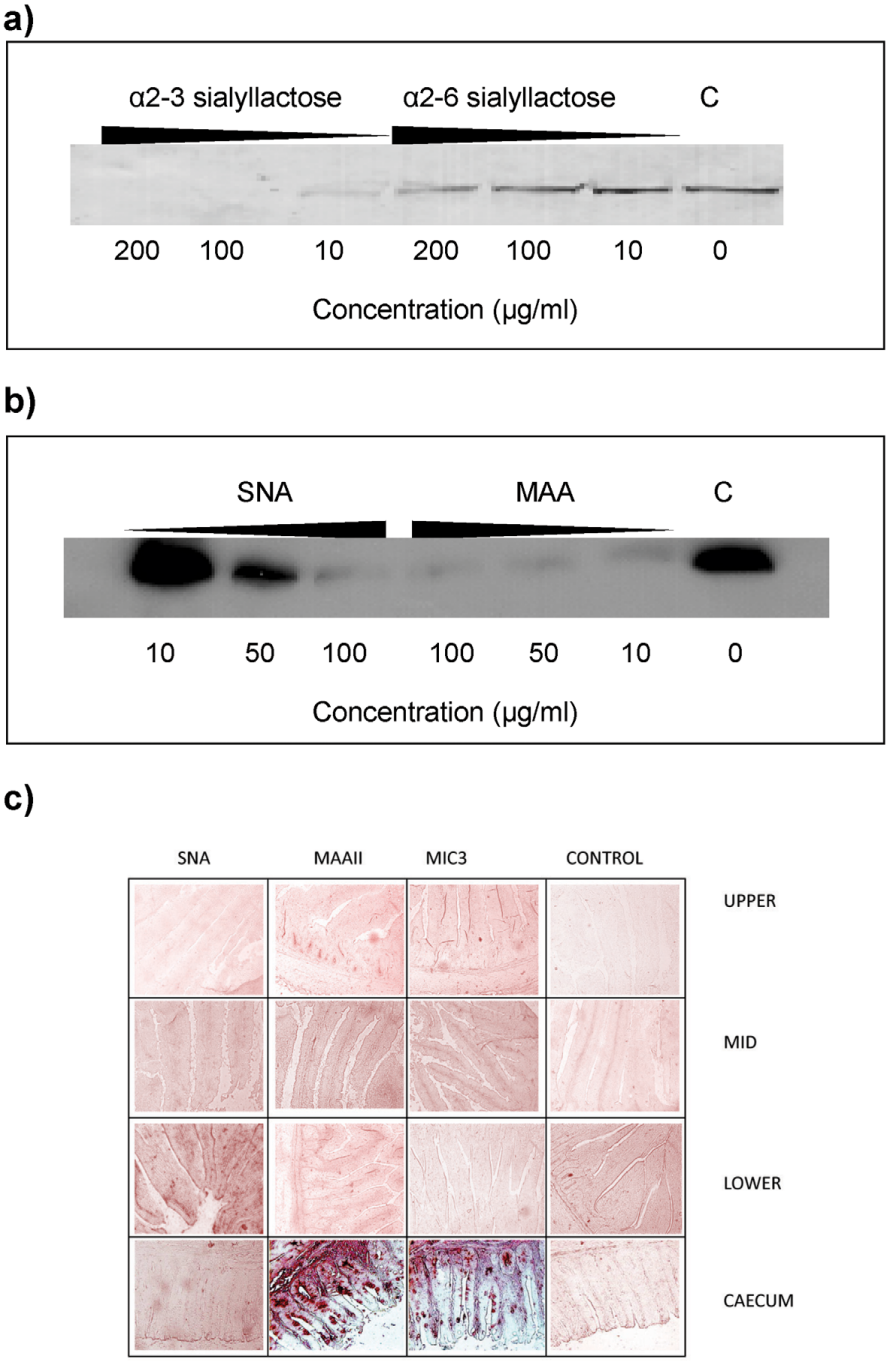
EtMIC3 targets sialic acid bearing glycans on host cells. (**a**) Western blot analysis of a cell bound fraction of EtMIC3 from a sporozoite lysate (20×10^6^/ml) in presence of increasing amounts of either α2–3 sialyllactose or α2–6 sialyllactose. (**b**) Western blot analysis of a cell bound fraction of EtMIC3 from a sporozoite lysate (20×10^6^/ml) in presence of increasing amounts of lectins from *Sambucus nigra* (SNA) or *Maackia amurensis* (MAA). (**c**) Histology analysis of carbohydrate presentation within chicken intestine. Alkaline phosphatase staining of histological sections of chicken intestinal tissue derived from the upper, mid and lower intestine and the caecum following incubation with plant lectins SNA or MAAII, with recombinant EtMIC3-MAR5 protein or with thioredoxin as a control protein (C). EtMIC3 and MAAII bind abundantly to epithelial cells of the caecum indicating that the preferred binding site of EtMIC3 is in the region of the intestine that expresses a high level of α2–3 sialylated glycans.

Sialyllactose with α2–3-linked sialic acid (Siaα2–3Galβ1–4Glc) was more effective in inhibiting the binding of EtMIC3 from a sporozoite lysate to MDBK cells than α2–6-linked sialyllactose (Figure 4a). Binding assays were also performed when MDBK cells were preincubated with plant lectins that have preferences for binding either α2–6 or α2–3 sialyl linkages, namely *Sambucus Nigra* agglutinin (SNA) [14] and *Maackia amurensis* agglutinin (MAA) [15], respectively. MAA (consisting of both *Maackia amurensis* hemagglutinin and leukoagglutinin) inhibited the binding of EtMIC3 to MDBK cells at lower concentrations compared with SNA (Figure 4b). This indicates that α2–3-linked sialyl glycans present on the MDBK cell surface are the dominate ligands in the EtMIC3 binding.

To test the cell specificity of EtMIC3 binding in host tissues, we performed binding assays using histological sections taken from throughout the chicken intestine (Figure 4c). Incubation of these sections with biotinylated SNA and *Maackia amurensis* hemagglutinin (MAAII) lectins showed an abundance of MAAII staining (and by inference, mucin-type sialyl glycan sequences [15]) in sections taken from caecum. The tissue staining pattern observed for EtMIC3 was similar to that of MAAII: the binding was predominantly to the caecal epithelium.

### EtMIC3 binds to a broad spectrum of oligosaccharides that terminate in sialic acid in carbohydrate microarrays

**Figure 5:**
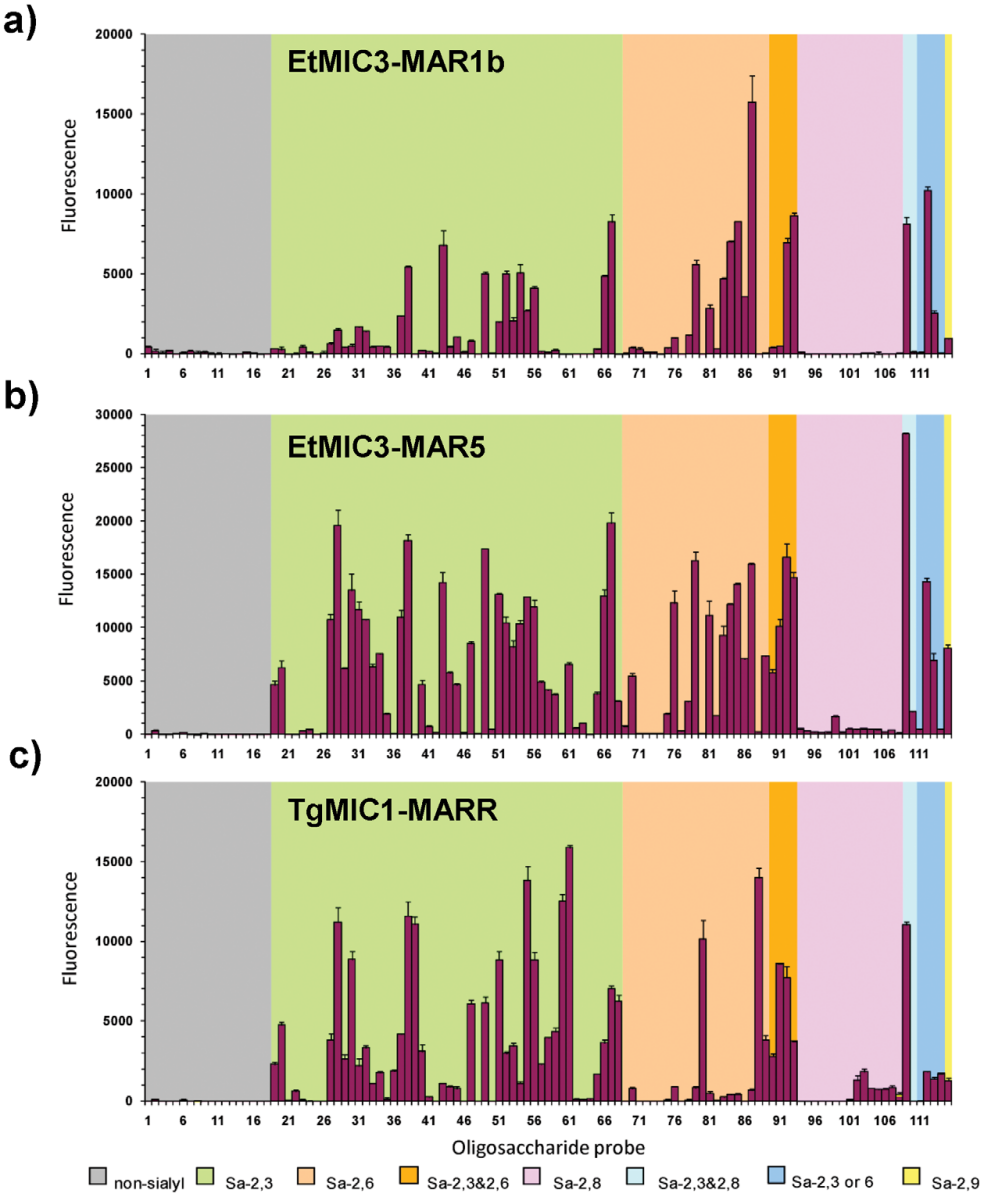
Carbohydrate microarray analyses of MAR domains. Carbohydrate microarray analyses of recombinant EtMIC3-MAR1b (a), EtMIC3-MAR5 (b) and TgMIC1-MARR (c) using microarrays of 115 lipid-linked oligosaccharide probes. Numerical scores of the binding signals are means of duplicate spots at 5 fmol/spot (with error bars). The various types of terminal sialic acid linkage are indicated by the coloured panels as defined at the bottom of the figure. The list of probes and their sequences and binding scores are in Table 1.

To assess the carbohydrate binding specificity of EtMIC3, we performed cell-independent binding analyses using carbohydrate microarrays composed of 115 lipid-linked oligosaccharide probes. Among these are 97 sialylated probes with differing sialic acid linkages, backbone chain lengths and sequences; 18 nonsialylated (neutral and sulfated) probes were included as negative controls (Figure 5, and Table S1). Microarray analyses were performed with recombinant proteins consisting of single MAR domain (EtMIC3-MAR1b), or tandem MAR domains (1a-1b-1d-1e, Figure S1) collectively referred to as EtMIC3-MAR5. For comparison we analyzed in parallel the recombinant MARR of *T. gondii* MIC1 (TgMIC1-MARR).

Similar to TgMIC1-MARR, the binding of EtMIC3-MAR1b and EtMIC3-MAR5 was to sialylated probes in the arrays, and no binding signal was observed with probes that lack sialic acids (Figure 5). Compared with the five-domain construct, EtMIC3-MAR5, the single domain EtMIC3-MAR1b had a more selective binding profile (Figure 5a). Notably, it gave little or no binding to sialyl di- or trisaccharide sequences, such as probes 19, 20, 27–34, 57–61, 70 and 76 (Table S1), but bound well to sialyl tetrasaccharides and longer sequences. Among the best ligands for EtMIC3-MAR1b are sialylated *N*-glycan probes (probes 56, 87 and 93), a sialyl Lewis^x^ (SiaLe^x^) probe which has a sulfate group at the 6-position of the *N*-acetylglucosamine (GlcNAc) residue (probe 49), and ganglioside-related probes that have terminal α2–3 sialic acid and side chain sialic acid, e.g. GD1a and GT1b (probes 66 and 109, Table 1). The five-domain construct, EtMIC3-MAR5 bound to a broader spectrum of sialyl probes with enhanced binding intensities overall compared to MAR1b (Figure 5a and b). Little or no binding was observed to α2–8-linked sialyl probes with the two EtMIC3 proteins. The intensities of binding signals elicited with α2–3 and α2–6-linked sialyl sequences sharing similar backbones and lipid moieties were comparable (Table 1). This pattern of binding is different from that observed in the inhibition studies where free oligosaccharides were used as inhibitors of the binding of EtMIC3 sporozoite lysate to MDBK cells, in which the α2–3-linked sialyllactose was a more potent inhibitor (Figure 4a).

**Table 1:** Comparison of the binding intensities elicited by selected glycan probes in carbohydrate microarrays with EtMIC3-MAR5 and TgMIC1-MARR.

Position	Structure		Fluorescence signal intensities		
			EtMIC3-MAR1b	EtMIC3-MAR5	TgMIC1-MARR
	***Selected α2–3-linked and α2–6-linked sialyl sequences***				
**20**	NeuAcα-3Galß-4Glc-AO		254	6,260	4,734
**70**	NeuAcα-6Galß-4Glc-AO		349	5,441	767
**37**	NeuAcα-3Galß-3GlcNAcß-3Galß-4Glc-DH		2,347	10,945	4,163
**79**	NeuAcα-6Galβ-4GlcNAcβ-3Galβ-4Glc-DH		5,570	16,300	834
**56**	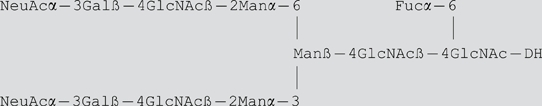		8,831	11,915	8,831
**87**	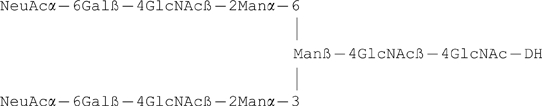		15,725	15,948	671
	***Selected fucosylated and sulphated sialyl sequences***				
**45**	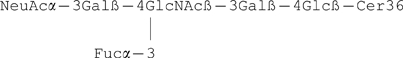	**(SiaLe^x^)**	1,031	4,623	785
**47**	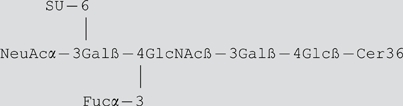	**(6'SU SiaLe^x^)**	790	8,527	6,077
**49**	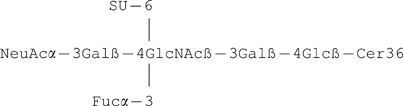	**(6SU SiaLe^x^)**	5,012	17,393	6,143
**51**	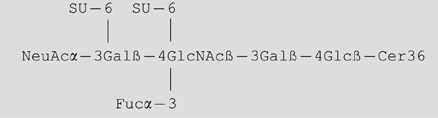	**(6,6'SU SiaLe^x^)**	1,997	13,131	8,834
	***Selected Ganglioside-related sequences***				
**66**	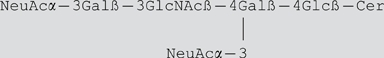	**(GD1a)**	4,849	12,980	3,630
**98**	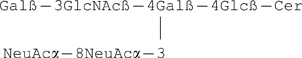	**(GD1b)**	-	217	-
**109**	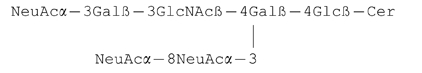	**(GT1b)**	8,121	28,174	11,032
**110**	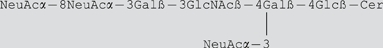	**(GT1a)**	88	2,114	-
	***Selected NeuAc and NeuGc-terminating sequences***				
**38**	NeuAcα-3Galβ-3GlcNAcβ-3Galβ-4Glcß-C30		5,423	18,142	11,580
**39**	NeuGcα-3Galβ-3GlcNAcβ-3Galβ-4Glcß-C30		-	-	11,075
**59**	NeuAcα-3Galß-4Glcß-Cer		232	3,694	4,327
**60**	NeuGcα-3Galß-4Glcß-Cer		-	-	12,526
**89**	NeuAcα-6GalNAc-AO		-	7,328	3,815
**88**	NeuGcα-6GalNAc-AO		47	264	13,982

-, less than 100.

A striking finding is that neither EtMIC3-MAR1b nor EtMIC3-MAR5 bound to sialyl sequences that terminate in the *N*-glycolyl form of sialic acids (NeuGc), e.g. probes 39, 60 and 88 in Table 1; this is in sharp contrast to TgMIC1-MARR which gave comparable or even stronger binding to the NeuGc probes than to their NeuAc analogs (Table 1).

### Structure of EtMIC3 MAR1 domain

To select a MAR domain for structural studies, each of the five unique MAR1 domains were analyzed for cell binding using a cell-based ELISA assay. All of the five MARRs, but not a thioredoxin control protein, bound to MDBK cells. The second, third and fourth MARR (MAR1b, 1c, 1d) exhibited the most intense binding signals (Figure S3). For high resolution structural analysis, we selected the second MAR domain, EtMIC3-MAR1b, which as shown above gave robust binding signals to sialyl glycan sequences in microarray analyses (Figure 5a).

**Figure 6:**
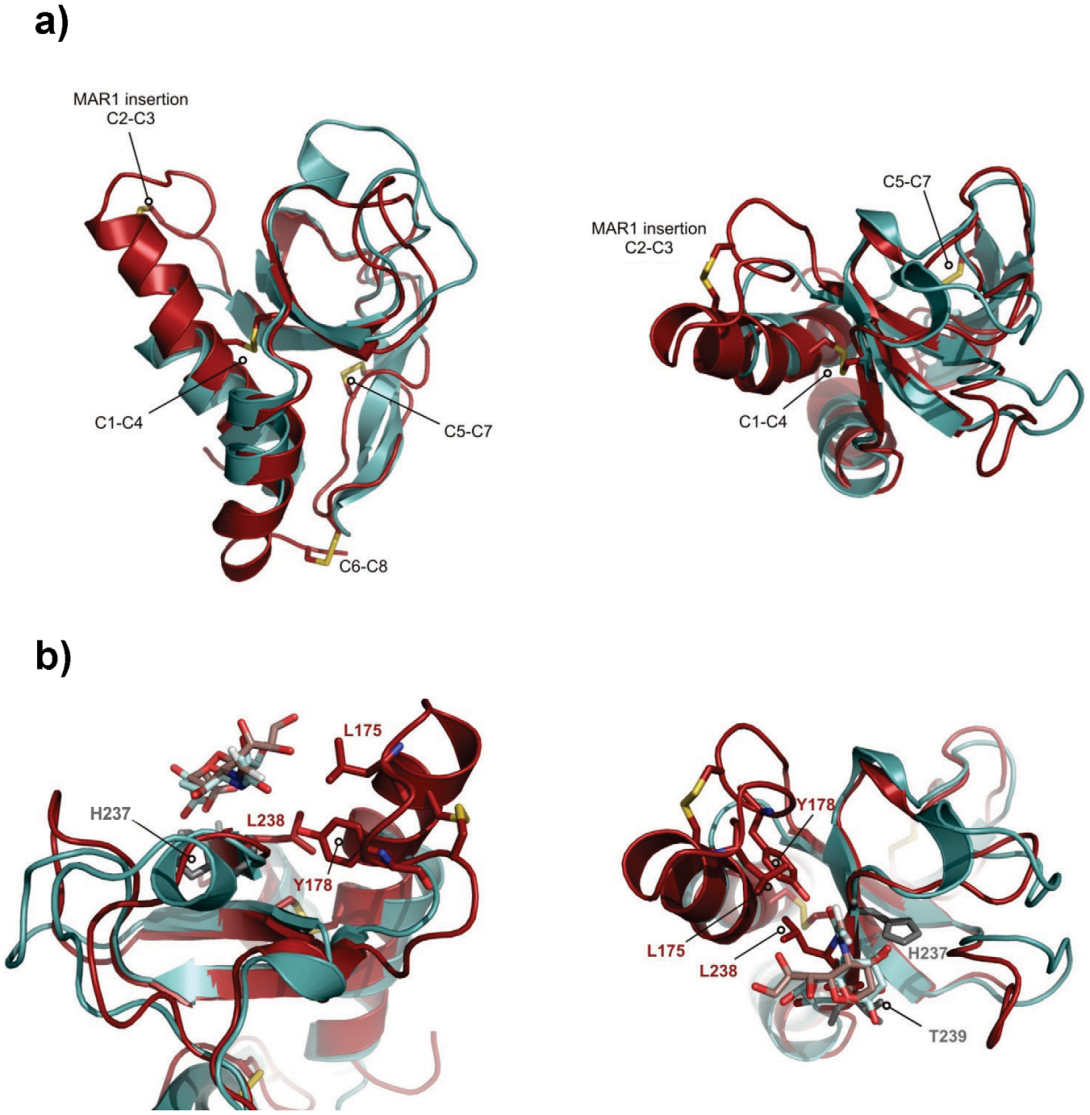
Three-dimensional structure of the MAR1b domain from EtMIC3. (**a**) Superposition of EtMIC3-MAR1b (red; PDB code 2LBO) on the MAR2 domain from TgMIC1 (PDB code 2JH1; cyan) showing the position of the disulfide bonds and ‘MAR1 insertion’ [4], [5]. Left and right images represent views from two orientations (Figure S1 and S4). (**b**) Superposition of EtMIC3-MAR1b (red; PDB code 2LBO) on the MAR2 domain from TgMIC1 (PDB code 2JH1; cyan) in complex with Siaα2–3Gal. For clarity only the sialic acid units are shown for both structures. Additional side-chain contacts from the MAR1 domain are shown in red. Conserved binding site HxT side chains are shown in purple. Left and right images represent views from two orientations (Figure S4).

The solution structure of EtMIC3-MAR1b determined by NMR spectroscopy comprises the distorted β-barrel arrangement and flanking helices of the classic MAR domain (Table 2). Three conserved disulfide bonds, C1–C4, C5–C7 and C6–C8 (namely C163–C201, C216–C226 and C220–C256, Figures 6a and S1) stabilize the core structure. A comparison of the structure with that of MAR1 and MAR2 of TgMIC1 reveals that EtMIC3-MAR1b contains a prominent extension to the first helix and subsequent loop (MAR1 insertion), which is stabilized and pinned together by an extra disulfide bond exclusive to the MAR1 subfamily (C2–C3 namely C171–C179; Figures 6a and S1). EtMIC3-MAR1b superimposes with an RMSD of 2.2 Å over 104 equivalent backbone C_α_ atoms with the MAR1 domain from TgMIC1 (PDB code 2JH1; Figure S4).

**Table 2:** Structural statistics from the solution structure calculation for EtMIC3.

Number of experimental restraints	(PDB code 2LBO)
Total NOE-derived	2918
Ambiguous	684
Unambiguous	1980
Intraresidue	738
Sequential	311
Medium-range (|i−j|≤4)	305
Long-range (|i−j|>4)	626
H-bonds	30
Talos (ϕ/ψ)	224
RMSD from experimental restraints	
Distance (Å)	0.055+/−0.003
Dihedral angle (deg.)	3.16+/−0.10

RMSD from idealized covalent geometry	
Bonds (Å)	0.0071+/−0.00013
Improper angles (deg.)	2.48+/−0.05
Angles (deg.)	0.81+/−0.02
Coordinate RMSD (Å)	
Backbone atoms in secondary structure	0.17+/−0.039
Heavy atoms in secondary structure	0.53+/−0.057
Ramachandran plot	
Residues in most favoured regions (%)	78.8
Residues in allowed regions (%)	18.9
Residues in disallowed regions (%)	0.4

The carbohydrate binding properties of the MAR2 subfamily have been well characterized structurally [4], [5], [6]; however no equivalent information is available for the MAR1 subfamily as occupancy in the MAR1 site of TgMIC1 was not established in soaking and co-crystallization experiments. Our NMR structure of EtMIC3-MAR1b provides an opportunity to study recognition of the MAR1 family in detail. To localize the carbohydrate binding region further, we performed NMR titration experiments using ^15^N, ^13^C-labeled EtMIC3-MAR1b in the presence of α2–3- and α2–6-linked sialyllactoses. Significant amide chemical shift changes were observed for resonances of several residues proximal to the expected carbohydrate binding site based on TgMIC1 (Figure S5). To provide an atomic resolution basis for recognition we embarked on the structural characterization for the carbohydrate-bound forms. Isotope ^13^C-filtered/edited NOESY spectra were recorded on complexes between ^13^C/^15^N-EtMIC3-MAR1b and sialyl *N*-acetyllactosamines (Siaα2–6Galβ1–4GlcNAc or Siaα2–3Galβ1–4GlcNAc) to identify intermolecular NOEs (Figure S4). A total of nine NOEs were assigned either to the ring protons or to unambiguously well dispersed side chains (Table S2). The α2–3 linked and α2–6 linked carbohydrate complexes exhibit similar patterns of intermolecular NOEs. Structures of the carbohydrate complexes were subsequently calculated invoking both intermolecular NOEs and chemical shift-derived distance restraints using the HADDOCK approach [16]. The lowest energy ensembles of water-refined structures superpose well over the intermolecular interface. As expected from NOEs the mode of sialic acid recognition is identical for the Siaα2–3Galβ1–4GlcNAc and Siaα2–6Galβ1–4GlcNAc, the major difference being the relative position of the galactose unit (Figure S4). Although the mode of recognition for the sialyl moiety is highly similar to that observed for MAR2 domain from TgMIC1, the ‘MAR1 insertion’ makes several new contacts via L175 and Y178 (Figure 6b).

### Sporozoite invasion of cultured epithelial cells can be inhibited by competitors of EtMIC3-sialic acid binding

**Figure 7:**
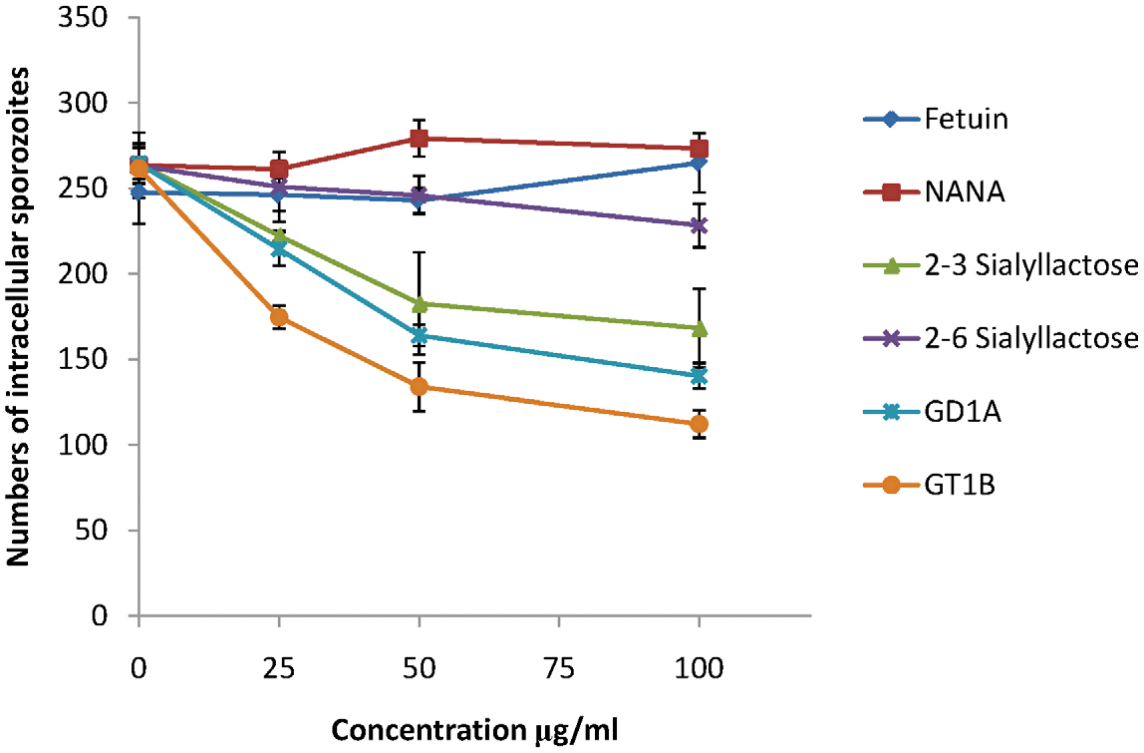
Sporozoite invasion of MDBK cells can be inhibited by sialic acid competitors. Freshly excysted sporozoites of *E. tenella* were incubated for 10 mins at room temperature with varying concentrations of sialylated molecules, and then allowed to invade semi-confluent monolayers of MDBK cells for 15 mins at 41°C. Cells were then fixed in methanol, stained in haematoxylin and eosin and the number of intracellular sporozoites enumerated.

MDBK is a cell line that supports invasion and intracellular development of *E. tenella* sporozoites and we used this to investigate whether sialylated structures on the cell surface contribute to parasite invasion. Using uracil uptake assays, in which parasite growth is measured over a period of 48 hours in culture, we found no significant reductions in radiolabel uptake following any of the treatments that had shown effects on EtMIC3 binding to MDBK cells (neuraminidase, fetuin, sialic acid, α2–3 or α2–6 sialyllactose, MAA or SNA lectins, gangliosides GD1a or GT1b). This indicates that binding to sialyl groups is not essential for overall parasite invasion of cultured cells. In these assays parasites were left in contact with the cells over the whole 48 hour period and as *in vitro* conditions differ so markedly from the *in vivo* situation, within the gut of the chicken, it is conceivable that specificity is swamped by other factors, especially over extended incubation periods. Given the immediate secretion of EtMIC3 and its likely deployment at very early during invasion, we next investigated the effect of these treatments on short-term cultures in which sporozoites were allowed to invade the MDBK monolayers for only 15 minutes before fixation and enumeration of intracellular parasites. Under these conditions we found dose-dependent inhibition of sporozoite invasion following treatments with GD1a and GT1b gangliosides and with α2–3 sialyllactose (Figure 7). In contrast treatment with fetuin, sialic acid or α2–6 sialyllactose did not cause significant inhibition of sporozoite invasion.

### Vaccination trials with EtMIC3 indicate its potential as a vaccine candidate

**Figure 8:**
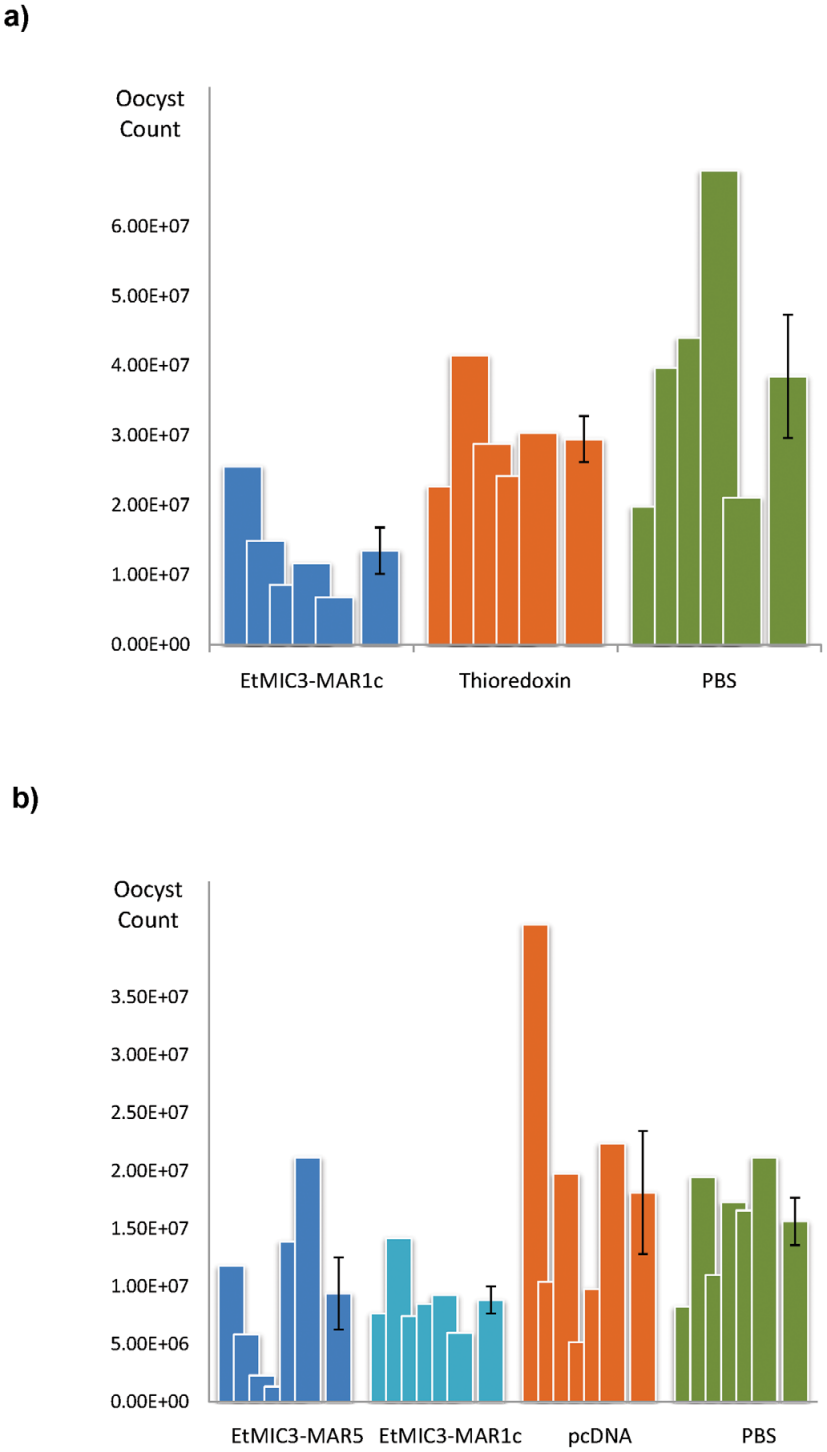
Vaccination trials with MARR from EtMIC3. (**a**) Counts for individual birds for each antigen are shown as overlapping columns and the average shown as a separate column with error bar. Immunization with recombinantly-expressed EtMIC3-MAR1c fusion protein resulted in an overall reduction in oocyst output after challenge of 54% compared to treatment with the thioredoxin fusion protein alone (See Table S3). (**b**) Counts for individual birds for each antigen are shown as overlapping columns and the average shown as a separate column with error bar. DNA immunization with pcDNA3.1 vector containing EtMIC3-MAR5 or EtMIC3-MAR1c resulted in an overall reduction in oocyst outputs after challenge of 48 and 51% respectively compared to treatment with the pcDNA3.1 vector alone (See Table S3).

We carried out exploratory immunization and challenge experiments in chicken to determine whether immunization with EtMIC3 could induce protection against parasite infection. Five independent experiments were carried out using purified EtMIC3-MAR recombinant protein (EtMIC3-MAR1c) or EtMIC3-MAR DNAs (of MAR1c and MAR5) as immunogens. In each case, following challenge with *E. tenella* oocysts, there was statistically significant reduction in oocyst shedding in vaccinated groups of birds compared to control groups (data from two independent experiments are shown in Figure 8).

## Discussion

Host cell invasion by apicomplexan parasites is a conserved and complex, multi-step process. While details are emerging of the key phases of invasion, the early stages remain the least well understood. It appears that parasites first attach transiently to the host cell surface via GPI-anchored surface proteins known in *T. gondii* as SRSs (Surface Antigen Glycoprotein Related Sequences) [17], [18] and which have been shown to bind sulfated glycosaminoglycans such as heparin [19], [20]. The loose attachment mediated by the SRSs is thought to enable efficient sampling of cell surfaces, which is followed by an irreversible interaction with the apical end of the parasite, allowing for proper engagement of the invasion machinery [2]. There are several adhesive microneme proteins that are neither assembled into the moving junction during invasion nor belong to the SRS family of surface proteins. The MARR-containing MIC proteins from coccidians are prominent examples of these and likely play a role in initiating the transition from transient, non-oriented binding to irreversible apical attachment.

We have demonstrated that EtMIC3, a MARR-containing protein from *Eimeria tenella*, is secreted at the early stages of invasion prior to the formation of the moving junction. It is localized at the interface of the host cell membrane and apical attachment of the parasite interface and remains proximal to the moving junction complex during invasion. EtMIC3 and EtAMA1 antibodies each label 14 focal points that decorate the circumference of the junction; the reason for this is unknown but it is unlikely to relate to sub-pellicular microtubules which number 24 in *E. tenella* (D. Ferguson, P. Monaghan and F. Tomley, unpublished observations). During the attachment stage of parasite invasion of MDBK cells, EtMIC3 was rapidly deployed to the sporozoite apical surface whereas another secreted microneme protein, EtMIC5, was not deployed. These observations raise the intriguing question as to whether the two molecules localize to the same or different populations of micronemes. Although heterogeneous populations of micronemes provides an attractive hypothesis for a staged deployment of microneme proteins during invasion, further work is required to provide further experimental evidence and elucidate the exact mechanism. Interestingly, invasion by *Eimeria tenella* is only sensitive to inhibition by soluble sialylated carbohydrates in the very early stages of contact between parasite and host cell (Figure 7). This observation is consistent with the view that EtMIC3 is secreted ahead of the moving junction to promote efficient cell-adhesion and assist in early stage invasion. This echoes the notion that parasites use multiple ligand–receptor interactions to ensure invasion during the various stages of the infection [21].

Compared to TgMIC1, a more restricted set of sialyl oligosaccharides are bound by EtMIC3; notably EtMIC3 does not recognize the *N*-glycolyl form of sialic acids and also shows little binding to α2–8-linked sialyl oligosaccharides. All of the MARR for EtMIC3 belong to the MAR1 family and three of these, MAR1b, MAR1c and MAR1d, are highly active for cell binding (Figure S3). In addition to active site TxH motif found in MAR2 domains, the MAR1 family also possess an extended first helix and loop which can be seen from the structure of EtMIC3-MAR1b to also coordinate the carbohydrate ligand via the side chains of L175 and Y178 (Figure 6b and S4). The methyl group of the *N*-acetyl moiety NeuAc makes several intimate contacts with this insertion, explaining why EtMIC3 cannot recognize the NeuGc form as the additional hydroxyl group would not be accommodated without significant rearrangement. Furthermore, the central residue in TxH is normally a small side chain in MAR2 domains, but in EtMIC3-MAR1b this is replaced by the larger leucine (i.e. L238) that contacts directly the glycerol side chain of sialic acid (Figure 6b and S4). The first and last MAR1 domains of EtMIC3 (MAR1a and MAR1e; Figure S1) show significant differences in these positions and this is consistent with the weaker binding of these two domains to cells (Figure S3).

Complex sialylated glycans are yet to be identified in coccidians [22], [23]. Given their wide distribution in warm-blooded animals, it is appropriate that sialylated glycans of the host are the targets for the MARR proteins. We have shown that binding specificity goes beyond simple recognition of sialic acid and includes the sialyl linkage, the chain length and further modification of the backbone sequence. Although stronger inhibitory activity was observed with α2–3-linked sialyllactose compared with the α2–6 analog in the inhibition of cell binding experiments, the preference for α2–3 sialyl oligosaccharides was not apparent in the carbohydrate microarray analyses. This is of interest, as in contrast to MAR1b and MAR5 of EtMIC3, the TgMIC1-MARR did show stronger binding to α2–3 than to α2–6 sialyl sequences in the microarrays (Figure 5c, and Table 1), in accord with our previous finding [4], [5].

With EtMIC3 there are maybe several explanations for the discrepancy in the relative potencies of sialyl α2–3 vs to α2–6 in the inhibition and the microarray binding assays. First, it is possible that the multivalent binding in the microarray system (both the oligosaccharide ligands and the EtMIC3 MAR domains are in oligomeric form) may result in substantial amplification of the binding response thus rendering it difficult to observe differential binding affinities/avidities towards the sialyl ligands. An example is the rather selective binding profile observed with the single-domain construct EtMIC3-MAR1b that becomes blurred with the five-domain construct EtMIC3-MAR5. It is also important to note that compared with the MAR2 domain present in TgMIC1, the EtMIC3 MAR domains (MAR1 family) have an inherent increased affinity for sialyl oligosaccharides due to additional interactions with sialic acid mediated by the MAR1 insertion between α1 and β1 and the H**L**T motif (Figures 6 and S1); this would make it more difficult to detect the difference in binding intensities for EtMIC3-MAR1b even when it was tested under the same conditions as TgMIC1-MARR. Second, the structure of the MAR2 domain of TgMIC1 revealed a water-mediated hydrogen bond network between protein (E205, E206 and E207) and Gal O6 position of the carbohydrate ligand, which contributed to the α2–3-linked sialyl oligosaccharide binding preference [4], [5]. Interestingly, several of these positions are replaced with other amino acid residues in the MAR1 domains from EtMIC3 (e.g. ^241^PSE in MAR1b, ^382^NPQ in MAR1c and ^533^NPQ in MAR1d); this may lead to the absence of structured water network thus providing an explanation for the lack of significant binding preference for α2–3-linked sialyl oligosaccharides in our microarray analyses. Finally, the increased flexibility of the α2–6 over the α2–3 sialyl linkage would also contribute to an additional entropic penalty upon ordering in the complex, and this may at least in part account for the stronger inhibitory activity of α2–3 sialyllactose in the inhibition assays where free oligosaccharides were used in solution as inhibitors.

*E. tenella* sporozoites invade primarily the caecal epithelium of chickens, in contrast to *T. gondii* zoites which have the ability to infect almost any nucleated cell. All the EtMIC3 MAR domains belong to the MAR1 family and they are likely to have similar binding specificities; whereas *T. gondii* possesses several proteins (e.g. TgMIC1 and TgMIC13) with diverse MAR domains (MAR1 and MAR2 family) that are capable of more promiscuous glycan binding.

It is interesting that our microarray analyses revealed a lack of recognition of NeuGc-terminating glycans by EtMIC3, which are rare in the chicken host [24]. The opposite, namely a preference of the NeuGc form of the sialyl ligand GM1, was revealed by microarray analysis of the oncogenic virus SV40 for which the primary host is the monkey that, unlike humans, can synthesize NeuGc [25]. The *T. gondii* MIC1 recognizes glycans with both NeuAc and NeuGc forms of sialic acids consistent with its very broad cell tropisms. These differing specificities are clearly major factors in the host tropisms of these microbes. The restricted tissue staining pattern observed for EtMIC3, namely in the chicken caecal epithelium, but not in other parts of the chicken intestine, indicates that EtMIC3 play a key role in efficiently directing the parasite to the caecum.

Apart from the specificities of EtMIC3-MAR1b toward different sialic acid forms and linkages, the carbohydrate microarray analyses have revealed modulation of binding strength in the presence of certain sulfate modifications of the sialyl oligosaccharide sequences. EtMIC3-MAR1b gave stronger binding to the 3'SiaLe^x^ sequence that has a sulfate at position 6 of the GlcNAc residue (6-SU SiaLe^x^; probe 49 in Table 1) than to analogs lacking sulfate on GlcNAc (probe 45 and 47) or having an additional sulfate group on the galactose (Gal) residue (6,6′-SU SiaLe^x^; probe 51). We have previously reported that sulfation pattern plays an important role in carbohydrate recognition by *Neospora caninum* MIC1 [6]. In that study, we observed strong binding of NcMIC1-MARR to two sulfated SiaLe^x^ probes both of which have a sulfate group on the Gal residue (as in probes 47 and 51). These properties of MIC proteins might have implications for tissue tropism. It is worth noting that the greater binding to 6-SU SiaLe^x^ sequence is a feature shared with highly pathogenic poultry influenza viruses including H5N1 viruses [26], [27]. These viruses also target the chicken intestinal tract.

Rotational treatment with anticoccidial drugs and commercial live vaccine is current best way to control infecting within chicken flocks. Due to the high expense of scaling-up the production of live parasite vaccine, there have been a number of recent efforts to develop subunit and recombinant coccidiosis vaccines using both DNA and protein based antigens [28], [29], [30], [31]. However, few have been successful and much work needs to be done to identify appropriate antigens and the optimal mode of delivery. The role of EtMIC3 in targeting host sialyl glycans in the early stages of invasion and its prominent location at the host-parasite interface suggests that it may serve as an effective vaccine antigen. We have carried out five independent challenge experiments in groups of birds vaccinated with recombinant EtMIC3-MAR protein or EtMIC3-MAR DNA and found that EtMIC3 vaccination results in highly significant reductions in oocyst output after challenge infection (Figure 8). Whilst these are small-scale experiments, the consistency of the trials and level of efficacy (around 50% reduction in oocyst shedding following vaccination) are higher than seen in many studies with other antigens, indicating that EtMIC3 should be considered as a good candidate antigen for future recombinant vaccine development.

## Materials and Methods

### Ethics statement

This study was carried out in strict accordance with the Animals (Scientific Procedures) Act 1986, an Act of Parliament of the United Kingdom. All animal studies and protocols were approved by the Institute for Animal Health Ethical Review Committee and the United Kingdom Government Home Office under project license number PPL 80/2545.

The authors are committed to the principals of the 3Rs: reduction (in numbers), refinement (of procedures) and replacement (with laboratory procedures) of experimental animals, commensurate with being able to do statistically and biologically significant experiments for animal health. Enriched environments have been introduced for our animals in line with guidelines from the National Centre for the 3Rs.

### Cloning, expression and purification from *E. coli*

Recombinant EtMIC3 fragments were expressed and purified using previously described strategies [32], [33]. Constructs corresponding to EtMIC3-MAR1a (residues 42 to 153), EtMIC3-MAR1b (residues 154 to 289), EtMIC3-MAR1c (residues 290 to 440) EtMIC3-MAR1d (residues 743 to 874), EtMIC3-MAR1e (residues 875 to 988) and EtMIC3-MAR5 (comprising sequences encompassing MAR1a, MAR1b, MAR1d and MAR1e residues 1-298,750-921) were each cloned into pET32b Xa/LIC plasmid (Novagen) and expressed as thioredoxin fusion proteins in Origami (DE3) (Novagen) [34]. For binding assays and vaccination experiments recombinant proteins were used as thioredoxin-hexa-His fusions and unfused thioredoxin protein was prepared in parallel from ‘empty’ vector as a control. For structure calculation an optimized construct of EtMIC3-MAR1b was generated encompassing residues 153–274. Protein expression was induced with 500 µM isopropyl β-D-thiogalactopyranoside overnight at 30°C. The fusion protein was purified by affinity chromatography using a nickel-nitrilotriacetic acid (Ni-NTA) resin (Qiagen) and separated from thioredoxin in a factor Xa cleavage reaction (Novagen). Factor Xa was removed from the sample by binding it to an immobilised Xarrest agarose resin (Novagen) and residual protease was inhibited with 1 mM AEBSF (Novagen). Thioredoxin was removed from the sample by passing the sample back across a Ni-NTA resin. The protein was concentrated to ∼1 mM and exchanged into phosphate buffer for NMR (20 mM sodium phosphate pH 6.5, 50 mM NaCl for the structure calculation and 20 mM sodium phosphate pH 5.5 mM NaCl for binding studies). ^15^N,^13^C-labelled samples were produced in minimal media, containing 0.07% ^15^NH_4_Cl and 0.2% ^13^C_6_-glucose. Recombinant TgMIC1-MARR was prepared as described [4], [6].

### Cell binding and inhibition experiments

Confluent monolayers of MDBK cells were blocked with 1% BSA in PBS for 2 h at 4°C, washed three times in PBS then incubated with sporozoite lysate or recombinant-expressed proteins (0.001 mg/ml to 2 mg/ml) for 1 h at 4°C. Monolayers were washed four times with PBS to remove unbound proteins, then cells and bound proteins were solubilised in SDS sample buffer, separated by SDS PAGE, transferred to nitrocellulose and probed with rabbit sera raised to EtMIC1, EtMIC2, EtMIC3 and EtMIC4. Dose dependent binding of EtMIC3 was determined by ELISA using gluteraldehyde fixed MDBK cells.

For binding inhibition experiments, MDBK cells were treated as follows: neuraminidase (from *C. perfringens*) obtained from Sigma, used at 0.5 to 0.125 units/ml, 37°C, 1 h; lectins SNA (from *Sambucus nigra*) or MAA (from *Maackia amurensis*) obtained from Sigma, used at 10, 50 or 100 µg/ml, 4°C for 30 min. Alternatively, sporozoite lysates or recombinant proteins were treated as follows: fetuin and asialofetuin (from fetal calf serum) obtained from Sigma, used at 1 to 1000 µg/ml, 4°C for 10 min; sialic acid (NeuAc), α2–3 sialyllactose and α2–6 sialyllactose (from bovine colostrum) obtained from Sigma, used at 10–200 µg/ml, 4°C for 10 min; gangliosides GD1a, GD1b and GT1b (from bovine brain), obtained from Sigma, ganglioside GD1a (from bovine brain) obtained from Alexis biochemicals), used at 100 µg/ml 4°C for 10 min.

### Carbohydrate microarray analysis

Microarrays were composed of lipid-linked oligosaccharide probes, namely neoglycolipids (NGL) and glycolipids, robotically printed on nitrocellulose-coated glass slides at 2 and 5 fmol per spot using a non-contact instrument [4], [35]. The NGLs were prepared by either reductive amination [36] or oxime ligation [37]. Among these are 97 sialylated probes with differing sialic acid linkage, glycan backbone, chain length and sequence, and 18 nonsialylated (neutral and sulfated) probes were included as negative controls (Figure 5, and Table S1). The microarray binding assays were performed at ambient temperature. His-tagged EtMIC3-MAR1b and TgMIC1-MARR were assayed essentially as described [4], [6]). In brief, the arrayed slides were blocked for 1 h with 1% w/v bovine serum albumin (Sigma) in Pierce Casein Blocker solution (casein/BSA). EtMIC3-MAR1b and TgMIC1-MARR were precomplexed with mouse monoclonal anti-polyhistidine and biotinylated goat anti-mouse IgG antibodies (Sigma) in a ratio of 1∶2.5∶2.5 (by weight) and overlaid onto the arrays at 40 µg/ml. For the analyses of EtMIC3-MAR5, precomplexation was not required. The protein was tested at 40 µg/ml, and followed by overlay with anti-polyhistidine and biotinylated anti-mouse IgG antibodies (10 µg/ml, precomplexed in a ratio of 1∶1). Binding was detected using Alexa Fluor 647-conjugated streptavidin (Molecular Probes). Microarray data analysis and presentation were carried out using dedicated software [38]. The binding to oligosaccharide probes was dose-related, and results at 5 fmol per spot are shown.

### Immunofluorescence assay (IFA) of *E. tenella* sporozoites and gut stages

*E. tenella* sporozoites were allowed to settle at ambient temperature onto monolayers of MDBK cells grown on coverslips, then these were incubated at 41°C for 5 min. Cells were fixed in 4% paraformaldehyde, permeabilized with Triton X-100 and blocked with 1% BSA in PBS. Caecae were removed from infected chickens at 96 h post infection and fixed in paraformaldehyde. Sections were submitted to histology for automated dehydration, paraffin embedding, sectioning and staining. They were cut using a microtome, dewaxed, pressure cooked and treated with 1% BSA in PBS to block non-specific staining [39] Cell monolayers and gut sections were incubated with various sera generated against *E. tenella* proteins as previously described [40]; chicken anti-EtMIC3 serum (1∶300), rabbit anti-EtMIC3 serum, (1∶300), mouse anti-tubulin (1∶1000), chicken anti-EtAMA1, (1∶400) or rabbit anti-EtMIC5 serum (1; 200). After washing, cells were incubated with appropriate secondary antibodies; goat anti-chicken-Alexa Fluor 488 (green) and goat anti–mouse Alexa Fluor 568 (red) and then briefly incubated with DAPI. Coverslips and sections were examined with a Zeiss Axioskop microscope or a Leica confocal microscope using Ar, Kr and 633 HeNe lasers.

### Transmission electron microscopy (TEM) of *E. tenella* sporozoites

Samples (pellets of sporozoites or pieces of infected tissue) were fixed in 2% paraformaldehyde in 0.1 M phosphate buffer, dehydrated and embedded in LR White resin. Thin sections were blocked with 1% BSA in PBS, floated on drops of rabbit anti-MIC3 antibody, washed and exposed to goat anti-rabbit Ig conjugated to 10 nm gold. Finally grids were washed and stained with uranyl acetate prior to examination in the electron microscope.

### NMR spectroscopy and structure calculation

All NMR spectra on the structure determinations of EtMIC3-MAR1b (residues 153–274) were recorded on ^15^N, ^13^C-labelled samples. Backbone and side-chain assignment were completed using standard double and triple-resonance assignment methodology [41], [42], [43]. The side-chain assignments were completed using HCCH-total correlation (TOCSY) spectroscopy and (H)CC(CO)NH TOCSY [43]. 3D ^1^H-^15^N/^13^C NOESY-HSQC (mixing time 100 ms at 500 MHz and 800 MHz) experiments provided the distance restraints used in the final structure calculation.

The ARIA protocol [44] was used for completion of the NOE assignment and the interface to the CNS structure calculation program [45]. Dihedral angle restraints derived from TALOS+ were also implemented [46]. The frequency window tolerance for assigning NOEs was ±0.05 ppm and ±0.07 ppm for direct and indirect proton dimensions and ±0.5 ppm and ±0.5 ppm for nitrogen and carbon dimensions, respectively. The ARIA parameters, p, Tv, and Nv, were set to default values. A slow cooling step was used with 72000 steps of 0.003 ps dynamics [47]. The 10 lowest energy structures had no NOE violations greater than 0.5 Å and dihedral angle violations greater than 5°. The structural statistics are presented in Table 1.

For the HADDOCK-derived structures of the carbohydrate-bound complexes the following protocol was used. The final family of 10 structures for EtMIC3-MAR1b were used as starting structures. The family of starting structures for the carbohydrate ligand were generated by selecting random torsion angles about the glycosidic bond. Two thousand starting structures for the complex were generated by selecting random structures from the above families and carrying out rigid-body minimisation, from which 1000 were used for subsequent simulated annealing (SA). During the SA and subsequent water-refinement stage, amino acid side chains within the putative carbohydrate binding site and whole carbohydrate ligand were allowed complete flexibility. The entire Raver1 peptide was also allowed complete flexibility during the calculation. 200 lowest-energy SA models were selected for a final water-refinement stage. NOE restraints were derived in standard fashion from heteronuclear-filtered NOE spectra (9 NOEs were identified in each complex and hydrogen bond restraints were included between the sialic acid carboxylate and threonine residue in the HLT motif). NOEs to sugar rings were implemented in an ambiguous manner.

### Chemical shift mapping for the EtMIC3-MAR1b and sialyl-containing carbohydrates

For NMR mapping experiments, ^15^N-labelled EtMIC3-MAR1b was prepared in 20 mM sodium phosphate buffer at pH 5.5 at approximately 1 mM in 0.5 ml. Either sialic acid (NeuAc), Siaα2–3Galβ1–4Glc, Siaα2–6Galβ1–4Glc, Siaα2–3Galβ1–4GlcNAc or Siaα2–6Galβ1–4GlcNAc in the same buffer were introduced at several steps up to a 50 fold molar excess and 2D ^1^H-^15^N HSQC spectra were recorded at each stage under identical experimental conditions. The final saturated position is shown in Figure S5.

### Chicken intestine histology

Samples of intestinal tissue including upper, mid, lower intestine and caeca, were removed immediately post-mortem from a 3 week old SPF Light Sussex chicken into 10% buffered formalin and subsequently embedded in paraffin. Sections (10 µm) were cut onto glass slides, dewaxed and treated with 10 mM Na citrate (pH 6.5) for 15 min in a microwave oven. Sections were blocked overnight in 5% BSA then binding of EtMIC3 or biotinylated plant lectins SNA or MAA-II (Vector Lab) was carried out using a Vectastain ABC-AP system. For EtMIC3, sections were incubated first with normal mouse serum, then rinsed and incubated with EtMIC3-MAR5 recombinant protein (100 µg/ml) for 30 min followed by washing, incubation with mouse anti-His serum for 30 min, washing and incubation with diluted biotinylated secondary antibody for 20 min. For SNA or MAAII, sections were incubated directly with biotinylated lectins (∼20 µg/ml; Vector Labs). All sections were processed for development with the VECTASTAIN ABC-AP reagent and substrate according to the manufacturer's instructions.

### Sporozoite invasion, replication and inhibition assays

*In vitro* infection of Madin Darby Bovine Kidney (MDCK) cells was carried out essentially as described previously [40]. Briefly for invasion assays, semi-confluent monolayers grown on coverslips in 24-well tissue culture plates were infected with 10^6^ freshly purified sporozoites and the plates re-incubated at 41°C for 15 minutes, at which time they were fixed in methanol, stained with haematoxylin and eosin, mounted under polyvinyl resin and examined at 400×magnification. The total number of intracellular parasites (within vacuoles) for 10 random fields was counted on each coverslip and at least three coverslips were examined for each treatment. For uracil uptake assays that measure parasite replication as an indirect read-out of parasite invasion [48], semi-confluent monolayers in 96-well plates were infected with 10^5^ freshly purified sporozoites together with 1 µCi [5,6]-[^3^H]-uracil (Perkin-Elmer NEN) and incubated for 48 hr, after which cells were lysed, harvested onto glass fibre filter mats with a cell harvester (Packard Filtermate) and uracil uptake quantified as counts per minutes using a direct beta-counter (MicroBeta, Perkin-Elmer-Wallac). Each treatment was replicated four times and control wells without parasites were set up for each experiment. Treatments used in this study were pre-incubation of sporozoites with fetuin, asialofetuin, sialic acid (NeuAc), α2–3 sialyllactose, α2–6 sialyllactose, gangliosides GD1a or GT1b (ranging from 10 µg/ml to 1 mg/ml) for 10 minutes at room temperature, or pre-incubation of MDBK cells with SNA or MAA lectins (ranging from 5 to 100 µg/ml) or neuraminidase (0.05 to 0.5 units/ml) for 1 hr at 41°C.

### Protection of chickens against challenge with *Emeria tenella*

Chickens were immunized with purified recombinant proteins (prepared as described above) or by DNA vaccination using pcDNA3.1 as the vector. Briefly, DNA corresponding to EtMIC3-MAR1c (residues 290–440) was PCR amplified with 5′ *Eco* RI and 3′ *Xba* I linkers and DNA corresponding to EtMIC3-MAR5 was PCR amplified with 5′ *Nco* I and 3′ *Hind* III linkers and each fragment cloned into pcDNA3.1.

One week-old pathogen free Light Sussex (SPF) chickens were divided into groups (n = 5–8). For protein immunizations, birds were injected subcutaneously with 100 µg recombinant protein split between two sites in the skin of the neck area. Three injections were administered at two weekly intervals, the first two in Titermax gold adjuvant (Sigma) and the third in Freund's incomplete adjuvant. Control groups were immunized with PBS or with thioredoxin fusion protein expressed and purified from empty pET32b vector. For DNA immunizations, birds were injected into the leg muscle with 100 µg plasmid split between two sites. Two injections were administered at two weekly intervals and control groups were immunized with PBS or with pcDNA3.1 plasmid DNA lacking a cloned insert. One week after the final immunization all birds were challenged with 250 *E. tenella* oocysts and total faecal droppings were collected from each individual bird on a daily basis from between 5 and 11 days post challenge. Faecal samples were processed to determine total oocyst counts from each individual bird using a MacMaster flotation chamber. Group averages and standard errors of the means were calculated and statistical significances between the means of different treatment groups determined using post-hoc Tukey analysis of variance.

## Supporting Information

Figure S1 **EtMIC3 Topology and MAR2 domain sequence alignment** (Top) Cartoon shows the location of each type of MARR in the full length EtMIC3 protein. (Bottom) Sequence alignments for the MAR domain families from EtMIC3 and TgMIC1. Cysteines are shaded orange and disulfide bond connectivities are indicated for the MAR domains. The position of the MAR1 insertion is shown by the arrows. Secondary structure elements are indicated above the sequence alignments; β-strands as arrows and α-helices as cylinders. Amino acid sequence numbers are indicated at the start and end of the rows.
(PDF)

Figure S2 **Inhibition of Cell binding of EtMIC3-MAR5.** Recombinant expressed protein (10 µg/ml) was incubated with either (1) fetuin -100 µg/ml (2) sialic acid -100 µgml (3) trisialoganglioside GT1a -100 µg/ml (4) disialoganglioside GD1a (Sigma) -100 µg/ml (5) disialoganglioside GD1b(Sigma) - 100 µg/ml, (6) disialoganglioside GD1a (Alexis biochemicals) -100 µg/ml or (7) no ligand control - 0 µg/ml, for 15 mins at 4°C and then incubated with MDBK cell monolayer for 15 min at 4°C. Monolayers were washed 3 times in PBS to remove unbound protein. The bound fraction was solubilized in SDS loading buffer and run on SDS PAGE gels, blotted and probed with ant-his antibody.
(PDF)

Figure S3 **Binding of individual MARR of EtMIC3 to fixed MDBK cell monolayers determined by ELISA.**
(PDF)

Figure S4 **Three-dimensional structure of the MAR1b domain from EtMIC3.** a) Stereo-view of the superimposition for the ten best NMR structures of EtMIC3-MAR1b. **b**) Superposition of EtMIC3-MAR1b (red; PDB code 2LBO) on the MAR2 domain from TgMIC1 (PDB code 2JH1; cyan). **c**) Superposition of EtMIC3-MAR1b (red; PDB code 2LBO) on the MAR1 domain from TgMIC1 (PDB code 2JH1; green). d) ^1^H-^13^C strips from filtered (^12^C, ^14^N)H-NOESY-^13^C-HSQC NMR experiment on ^13^C/^15^N-labelled EtMIC3-MARb in complex with Siaα2–6Gal. **e**) NMR-derived solution structure of EtMIC3-MAR1b in complex with Siaα2–6Gal. **f**) ^1^H-^13^C strips from filtered (^12^C, ^14^N)H-NOESY-^13^C-HSQC NMR experiment on ^13^C/^15^N-labelled EtMIC3-MARb in complex with Siaα2–3Gal. g) NMR-derived solution structure of EtMIC3-MAR1b in complex with Siaα2–3Gal.
(PDF)

Figure S5 **Chemical shift mapping for the interaction of recombinant EtMIC3-MAR1b with α2–6 sialyllactose.** (Top) ^1^H-^15^N HSQC spectrum for ^15^N,^13^C-labelled EtMIC3-MARb alone (black) and in presence of unlabelled Siaα2–6Galβ1–4Glc (pink) at a molar ratio of 1∶1. (Bottom) Surface representation for the lowest energy structure for EtMIC3-MARb with residues colour red according to the extent of chemical shift perturbation in the presence of Siaα2–6Galβ1–4Glc. Orientation is the same as in Figure 6.
(PDF)

Table S1 Oligosaccharide probes included in the microarrays sorted by sialyl linkage and backbone sequences, and the binding signals (fluorescence intensities) they elicited* with EtMIC3-MAR5 and TgMIC1-MARR.
(PDF)

Table S2 **Intermolecular NOE restraints for HADDOCK docking calculations of EtMIC3 with sialyl carbohydrates.**
(PDF)

Table S3 **Cumulative faecal oocysts counts from each individual bird in the two vaccination/challenge experiments shown in** **Figure 7** **.**
(PDF)
