# Supplementary figures and images for: The Role of Sialyl Glycan Recognition in Host Tissue Tropism of the Avian Parasite Eimeria tenella
Source: PLoS Pathog. 2011 Oct 13;7(10):e1002296. doi: 10.1371/journal.ppat.1002296 (PMC3192848; doi:10.1371/journal.ppat.1002296)

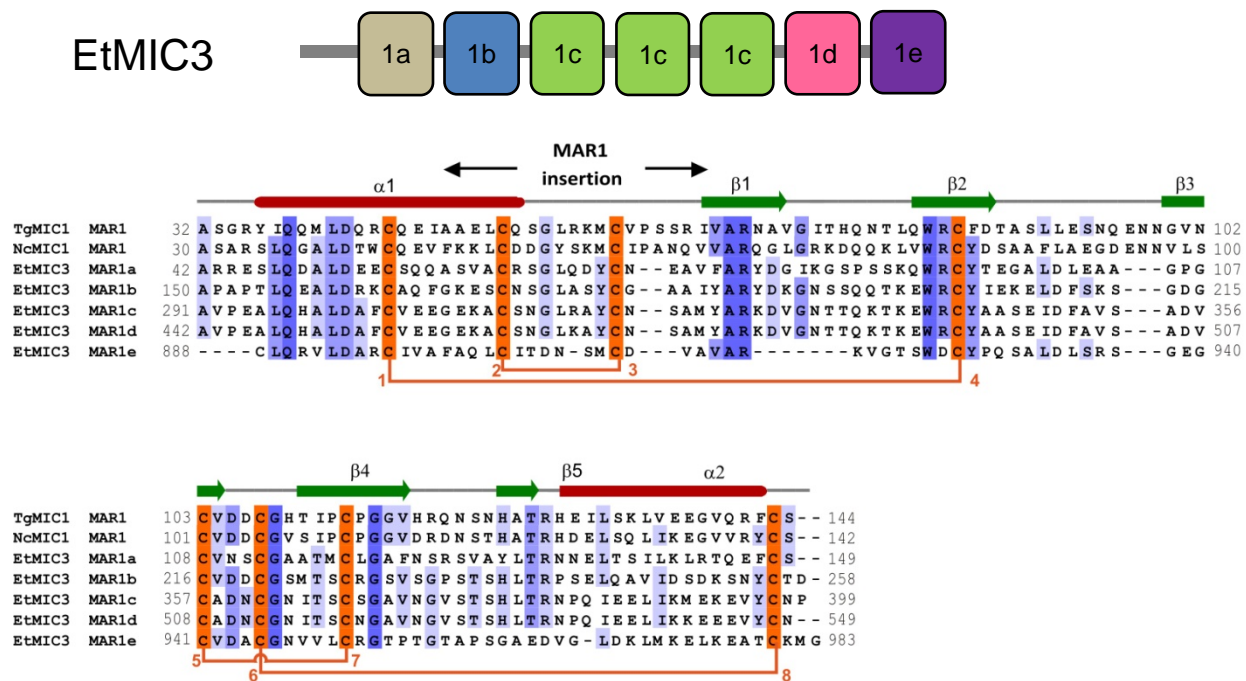

**Figure S1**

Supplement: Figure S1 — EtMIC3 Topology and MAR2 domain sequence alignment (Top) Cartoon shows the location of each type of MARR in the full length EtMIC3 protein. (Bottom) Sequence alignments for the MAR domain families from EtMIC3 and TgMIC1. Cysteines are shaded orange and disulfide bond connectivities are indicated for the MAR domains. The position of the MAR1 insertion is shown by the arrows. Secondary structure elements are indicated above the sequence alignments; β-strands as arrows and α-helices as cylinders. Amino acid sequence numbers are indicated at the start and end of the rows. (PDF) [file ppat.1002296.s001.pdf]

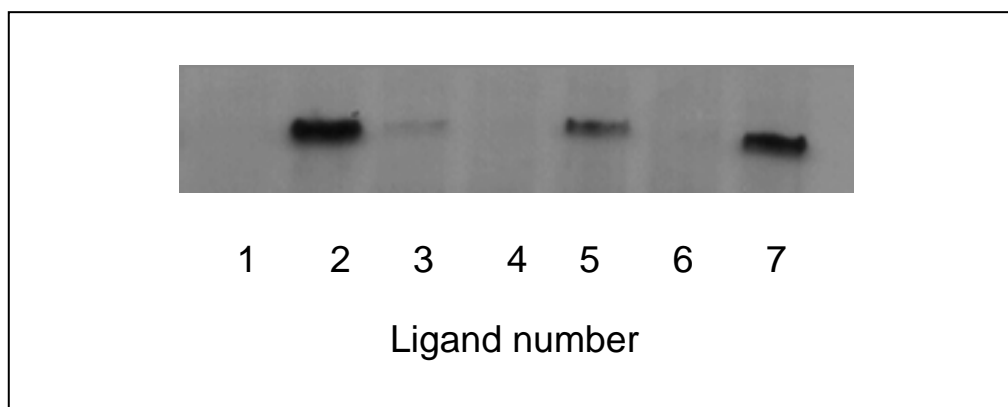

**Figure S2**

Supplement: Figure S2 — Inhibition of Cell binding of EtMIC3-MAR5. Recombinant expressed protein (10 µg/ml) was incubated with either (1) fetuin -100 µg/ml (2) sialic acid -100 µgml (3) trisialoganglioside GT1a -100 µg/ml (4) disialoganglioside GD1a (Sigma) -100 µg/ml (5) disialoganglioside GD1b(Sigma) - 100 µg/ml, (6) disialoganglioside GD1a (Alexis biochemicals) -100 µg/ml or (7) no ligand control - 0 µg/ml, for 15 mins at 4°C and then incubated with MDBK cell monolayer for 15 min at 4°C. Monolayers were washed 3 times in PBS to remove unbound protein. The bound fraction was solubilized in SDS loading buffer and run on SDS PAGE gels, blotted and probed with ant-his antibody. (PDF) [file ppat.1002296.s002.pdf]

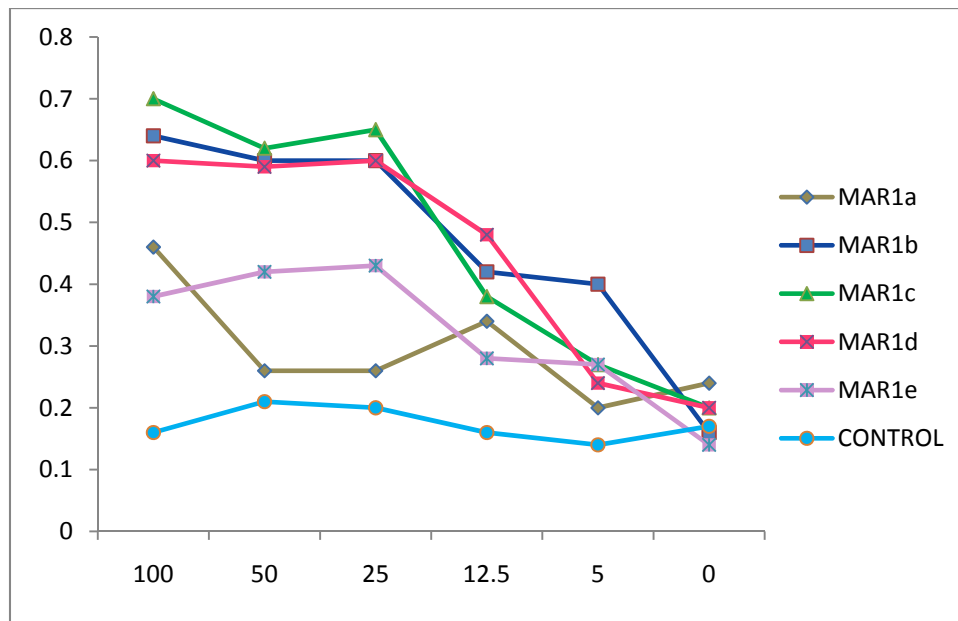

**Figure S3**

Supplement: Figure S3 — Binding of individual MARR of EtMIC3 to fixed MDBK cell monolayers determined by ELISA. (PDF) [file ppat.1002296.s003.pdf]

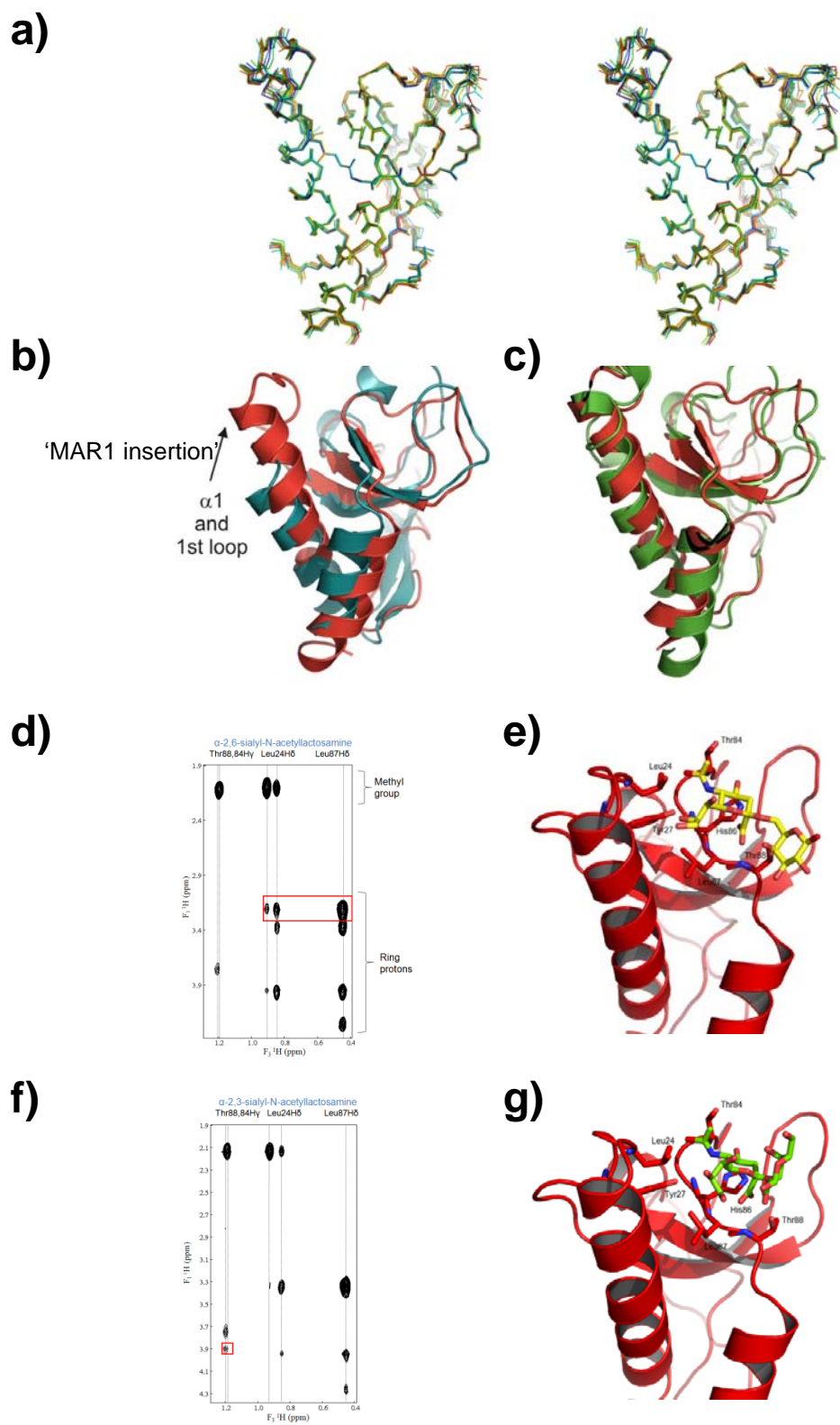

**Figure S4**

Supplement: Figure S4 — Three-dimensional structure of the MAR1b domain from EtMIC3. a) Stereo-view of the superimposition for the ten best NMR structures of EtMIC3-MAR1b. b) Superposition of EtMIC3-MAR1b (red; PDB code 2LBO) on the MAR2 domain from TgMIC1 (PDB code 2JH1; cyan). c) Superposition of EtMIC3-MAR1b (red; PDB code 2LBO) on the MAR1 domain from TgMIC1 (PDB code 2JH1; green). d) 1H-13C strips from filtered (12C, 14N)H-NOESY-13C-HSQC NMR experiment on 13C/15N-labelled EtMIC3-MARb in complex with Siaα2–6Gal. e) NMR-derived solution structure of EtMIC3-MAR1b in complex with Siaα2–6Gal. f) 1H-13C strips from filtered (12C, 14N)H-NOESY-13C-HSQC NMR experiment on 13C/15N-labelled EtMIC3-MARb in complex with Siaα2–3Gal. g) NMR-derived solution structure of EtMIC3-MAR1b in complex with Siaα2–3Gal. (PDF) [file ppat.1002296.s004.pdf]

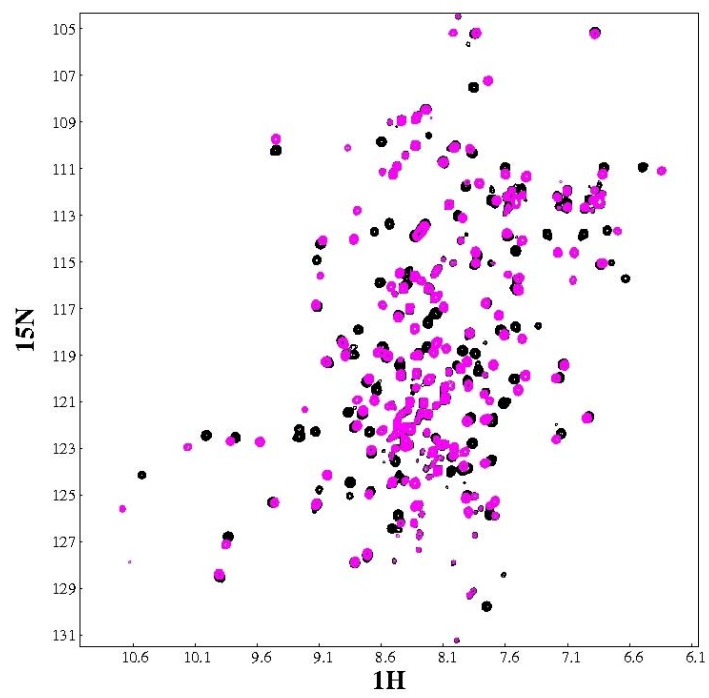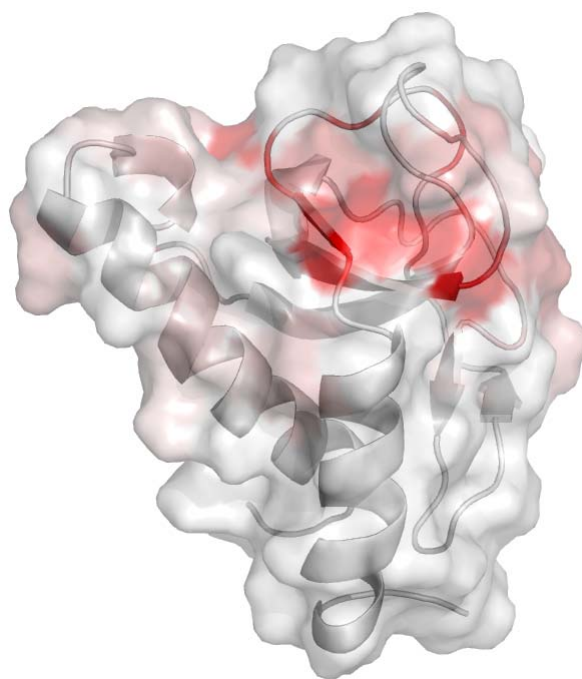

**Figure S5**

Supplement: Figure S5 — Chemical shift mapping for the interaction of recombinant EtMIC3-MAR1b with α2–6 sialyllactose. (Top) 1H-15N HSQC spectrum for 15N,13C-labelled EtMIC3-MARb alone (black) and in presence of unlabelled Siaα2–6Galβ1–4Glc (pink) at a molar ratio of 1∶1. (Bottom) Surface representation for the lowest energy structure for EtMIC3-MARb with residues colour red according to the extent of chemical shift perturbation in the presence of Siaα2–6Galβ1–4Glc. Orientation is the same as in Figure 6. (PDF) [file ppat.1002296.s005.pdf]
