# Supplementary material for: The Role of Sialyl Glycan Recognition in Host Tissue Tropism of the Avian Parasite Eimeria tenella
Source: PLoS Pathog. 2011 Oct 13;7(10):e1002296. doi: 10.1371/journal.ppat.1002296 (PMC3192848; doi:10.1371/journal.ppat.1002296)
Supplement: Table S1 — Oligosaccharide probes included in the microarrays sorted by sialyl linkage and backbone sequences, and the binding signals (fluorescence intensities) they elicited* with EtMIC3-MAR5 and TgMIC1-MARR. (PDF) [file ppat.1002296.s006.pdf]

Oligosaccharide probes included in the microarrays sorted by sialyl linkage and backbone sequences, and the binding signals (fluorescence intensities) they elicited* with EtMIC3-MAR5 and TgMIC1-MARR.

Position	Probe designation**	Sequence	EtMIC3-MAR1b	EtMIC3-MAR5	TgMIC1-MARR
**Non-sialylated (neutral and sulphated)**					
**1**	**Lac**	Galß-4Glc-DH	399	-	-
**2**	**Lac-AO**	Galß-4Glc-AO	183	336	127
**3**	**LacNAc(1-3)**	Galß-3GlcNAc-DH	6	-	-
**4**	**LacNAc(1-3)-AO**	Galß-3GlcNAc-AO	184	-	-
**5**	**LacNAc**	Galß-4GlcNAc-DH	-	31	-
**6**	**LacNAc-AO**	Galß-4GlcNAc-AO	73	179	54
**7**	**LNT**	Galß-3GlcNAcß-3Galß-4Glc-DH	182	-	-
**8**	**LNnT**	Galß-4GlcNAcß-3Galß-4Glc-DH	79	-	-
**9**	**Paragloboside**	Galß-4GlcNAcß-3Galß-4Glcß-Cer	83	33	-
**10**	**LNFP-II**	Galß-3GlcNAcß-3Galß-4Glc-DH<br>| Fucα-4	-	-	-
**11**	**LNFP-III**	Galß-4GlcNAcß-3Galß-4Glc-DH<br>| Fucα-3	-	-	-
**12**	**SU(3')-LNFP-II**	SU-3Galß-3GlcNAcß-4Galß-4Glc-DH<br>| Fucα-4	-	-	-
**13**	**SU(6')-LNFP-II**	SU-6Galß-3GlcNAcß-3Galß-4Glc-DH<br>| Fucα-4	-	-	-
**14**	**SU(3')-LNFP-III**	SU-3Galß-4GlcNAcß-3Galß-4Glc-DH<br>| Fucα-3	-	-	-
**15**	**SU(6')-LNFP-III**	SU-6Galß-4GlcNAcß-3Galß-4Glc-DH<br>| Fucα-3	92	-	-
**16**	**SU(3',6)-LNFP-III**	SU-6<br>| SU-3Galß-4GlcNAcß-3Galß-4Glc-DH<br>| Fucα-3	8	-	-
**17**	**Man9GN2**	Manα-2Manα-6<br>| Manα-2Manα-3Manα-6<br>| Manß-4GlcNAcß-4GlcNAc-DH<br>| Manα-2Manα-2Manα-3	-	-	-
**18**	**NA2**	Galß-4GlcNAcß-2Manα-6<br>| Manß-4GlcNAcß-4GlcNAc-DH<br>| Galß-4GlcNAcß-2Manα-3	-	-	-

2-3-linked sialyl					
19	NeuAcα-(3')Lac	NeuAcα-3Galß-4Glc-DH	317	4,635	2,298
20	NeuAcα-(3')Lac-AO	NeuAcα-3Galß-4Glc-AO	254	6,260	4,734
21	Neu4,5Ac-(3')Lac	Neu4,5Acα-3Galß-4Glc-DH	-	-	23
22	Neu4,5Ac-(3')Lac-AO	Neu4,5Acα-3Galß-4Glc-AO	-	-	621
23	Neuα-(3')Lac	Neuα-3Galß-4Glc-DH	398	326	74
24	Neuα-(3')Lac-AO	Neuα-3Galß-4Glc-AO	80	437	-
25	NeuAcß-(3')Lac	NeuAcß-3Galß-4Glc-DH	-	-	-
26	NeuAcß-(3')Lac-AO	NeuAcß-3Galß-4Glc-AO	76	6	-
27	NeuAcα-(3')LN1-3	NeuAcα-3Galß-3GlcNAc-DH	628	10,717	3,820
28	NeuAcα-(3')LN1-3-AO	NeuAcα-3Galß-3GlcNAc-AO	1,464	19,612	11,214
29	NeuAcα-(3')LN	NeuAcα-3Galß-4GlcNAc-DH	400	6,175	2,628
30	NeuAcα-(3')LN-AO	NeuAcα-3Galß-4GlcNAc-AO	461	13,499	8,872
31	SA(3')-Lea-Tri	NeuAcα-3Galß-3GlcNAc-DH<br>| Fucα-4	1,675	11,667	2,207
32	SA(3')-Lea-Tri-AO	NeuAcα-3Galß-3GlcNAc-AO<br>| Fucα-4	1,399	10,736	3,304
33	SA(3')-Lex-Tri	NeuAcα-3Galß-4GlcNAc-DH<br>| Fucα-3	420	6,342	1,117
34	SA(3')-Lex-Tri-AO	NeuAcα-3Galß-4GlcNAc-AO<br>| Fucα-3	491	7,566	1,795
35	GSC-105	NeuAcα-3Galß-4GlcNAcß-3Galß-Cer36<br>| Fucα-3	434	1,861	132
36	GSC-177	NeuGcα-3Galβ-4GlcNAcβ-3Galß-Cer36<br>| Fucα-3	-	33	1,899
37	LSTa	NeuAcα-3Galß-3GlcNAcß-3Galß-4Glc-DH	2,347	10,945	4,163
38	GSC-272	NeuAcα-3Galβ-3GlcNAcβ-3Galβ-4Glcß-C30	5,423	18,142	11,580
39	GSC-396	NeuGcα-3Galβ-3GlcNAcβ-3Galβ-4Glcß-C30	-	-	11,075
40	Sialylparagloboside	NeuAcα-3Galß-4GlcNAcß-3Galß-4Glcß-Cer	193	4,687	3,094
41	GSC-31	NeuAcα-3Galß-4GlcNAcß-3Galß-4Glcß-Cer36	135	747	259
42	GSC-516B	Neuα-3Galß-4GlcNAcß-3Galß-4Glcß-Cer36<br>| SU-6	55	189	-
43	SA(3')-LNFP-II	NeuAcα-3Galß-3GlcNAcß-3Galß-4Glc-DH<br>| Fucα-4	6,765	14,222	1,129

**44**	**SA(3')-LNFP-III**	NeuAcα-3Galß-4GlcNAcß-3Galß-4Glc-DH<br>| Fucα-3	438	5,750	891
**45**	**GSC-64**	NeuAcα-3Galß-4GlcNAcß-3Galß-4Glcß-Cer36<br>| Fucα-3	1,031	4,623	785
**46**	**GSC-472**	Neuα-3Galß-4GlcNAcß-3Galß-4Glcß-Cer36<br>| Fucα-3	118	119	-
**47**	**GSC-268**	SU-6<br>| NeuAcα-3Galß-4GlcNAcß-3Galß-4Glcß-Cer36<br>| Fucα-3	790	8,527	6,077
**48**	**GSC-268 deNAc**	SU-6<br>| Neuα-3Galß-4GlcNß-3Galß-4Glcß-Cer36<br>| Fucα-3	-	57	-
**49**	**GSC-269**	SU-6<br>| NeuAcα-3Galß-4GlcNAcß-3Galß-4Glcß-Cer36<br>| Fucα-3	5,012	17,393	6,143
**50**	**GSC-406**	SU-6<br>| Neuα-3Galß-4GlcNAcß-3Galß-4Glcß-Cer36<br>| Fucα-3	65	466	-
**51**	**GSC-270**	SU-6 SU-6<br>| | NeuAcα-3Galß-4GlcNAcß-3Galß-4Glcß-Cer36<br>| Fucα-3	1,997	13,131	8,834
**52**	**MSMFLNH**	Galß-4GlcNAcß-6<br>| | Fucα-3 Galß-4Glc-DH<br>| NeuAcα-3Galß-3GlcNAcß-3	5,008	10,442	3,007
**53**	**GSC-221**	NeuAcα-3Galß-4GlcNAcß-3Galß-4GlcNAcß-3Galß-4Glcß-Cer36<br>| Fucα-3	2,035	8,208	3,431
**54**	**GSC-220**	NeuAcα-3Galß-4GlcNAcß-3Galß-4GlcNAcß-3Galß-4Glcß-Cer36<br>| | Fucα-3 Fucα-3	5,049	10,350	1,129
**55**	**C4U**	NeuAcα-3Galβ-4GlcNAcβ-3Galβ-3GlcNAc<br>| | | SU-6 SU-6 SU-6	2,693	12,877	13,830
**56**	**A2F(2-3)**	NeuAcα-3Galß-4GlcNAcß-2Manα-6 Fucα-6<br>| | Manß-4GlcNAcß-4GlcNAc<br>| NeuAcα-3Galß-4GlcNAcß-2Manα-3	4,092	11,915	8,831
**57**	**GM4**	NeuAcα-3Galβ-Cer	153	4,859	2,299
**58**	**Haematoside**	NeuAcα-3Galß-4Glcß-Cer	97	4,167	3,959
**59**	**GM3**	NeuAcα-3Galß-4Glcß-Cer	232	3,694	4,327
**60**	**GM3(Gc)**	NeuGcα-3Galß-4Glcß-Cer	-	64	12,526
**61**	**GM2**	GalNAcβ-4Galβ-4Glcβ-Cer<br>| NeuAcα-3	-	6,547	15,875

<table>
<tr><td>62</td><td>GM1</td><td>Galβ-3GalNAcβ-4Galβ-4Glcβ-Cer
|
NeuAcα-3</td><td>-</td><td>587</td><td>117</td></tr>
<tr><td>63</td><td>GM1-penta</td><td>Galβ-3GalNAcβ-4Galβ-4Glc-DH
|
NeuAcα-3</td><td>-</td><td>1,051</td><td>102</td></tr>
<tr><td>64</td><td>GM1(Gc)</td><td>Galβ-3GalNAcβ-4Galβ-4Glcβ-Cer
|
NeuGcα-3</td><td>-</td><td>-</td><td>147</td></tr>
<tr><td>65</td><td>GM1(Gc)-penta</td><td>Galβ-3GalNAcβ-4Galβ-4Glc-DH
|
NeuGcα-3</td><td>302</td><td>3,762</td><td>1,691</td></tr>
<tr><td>66</td><td>GD1a</td><td>NeuAcα-3Galß-3GalNAcß-4Galß-4Glcß-Cer
|
NeuAcα-3</td><td>4,849</td><td>12,980</td><td>3,630</td></tr>
<tr><td>67</td><td>GD1a-hexa</td><td>NeuAcα-3Galß-3GalNAcß-4Galß-4Glc-DH
|
NeuAcα-3</td><td>8,240</td><td>19,790</td><td>7,031</td></tr>
<tr><td>68</td><td>GalNAc-GD1a(Ac,Gc)</td><td>GalNAcß-4Galß-3GalNAcß-4Galß-4Glcß-Cer
| |
NeuGcα-3 NeuAcα-3

GalNAcß-4Galß-3GalNAcß-4Galß-4Glcß-Cer
| |
NeuAcα-3 NeuGcα-3</td><td>-</td><td>3,113</td><td>6,247</td></tr>
<tr><td colspan="6">2-6-linked sialyl</td></tr>
<tr><td>69</td><td>NeuAcα-(6')Lac</td><td>NeuAcα-6Galß-4Glc-DH</td><td>-</td><td>752</td><td>-</td></tr>
<tr><td>70</td><td>NeuAcα-(6')Lac-AO</td><td>NeuAcα-6Galß-4Glc-AO</td><td>349</td><td>5,441</td><td>767</td></tr>
<tr><td>71</td><td>Neuα-(6')Lac</td><td>Neuα-6Galß-4Glc-DH</td><td>264</td><td>54</td><td>-</td></tr>
<tr><td>72</td><td>Neuα-(6')Lac-AO</td><td>Neuα-6Galß-4Glc-AO</td><td>118</td><td>78</td><td>-</td></tr>
<tr><td>73</td><td>NeuAcß-(6')Lac</td><td>NeuAcß-6Galß-4Glc-DH</td><td>89</td><td>8</td><td>-</td></tr>
<tr><td>74</td><td>NeuAcß-(6')Lac-AO</td><td>NeuAcß-6Galß-4Glc-AO</td><td>-</td><td>4</td><td>-</td></tr>
<tr><td>75</td><td>NeuAcα-(6')LN</td><td>NeuAcα-6Galß-4GlcNAc-DH</td><td>344</td><td>1,863</td><td>79</td></tr>
<tr><td>76</td><td>NeuAcα-(6')LN-AO</td><td>NeuAcα-6Galß-4GlcNAc-AO</td><td>976</td><td>12,298</td><td>906</td></tr>
<tr><td>77</td><td>Neu5,9Ac-(6')LN</td><td>Neu5,9Acα-6Galß-4GlcNAc-DH</td><td>-</td><td>293</td><td>-</td></tr>
<tr><td>78</td><td>LSTb</td><td>Galβ-3GlcNAcβ-3Galβ-4Glc-DH
|
NeuAcα-6</td><td>1,144</td><td>3,069</td><td>63</td></tr>
<tr><td>79</td><td>LSTc</td><td>NeuAcα-6Galβ4-GlcNAcβ3-Galβ4-Glc-DH</td><td>5,570</td><td>16,300</td><td>834</td></tr>
<tr><td>80</td><td>GSC-397</td><td>NeuGcα-6Galβ-3GlcNAcβ-3Galβ-4Glcß-C30</td><td>-</td><td>13</td><td>10,120</td></tr>
<tr><td>81</td><td>SA(6')-LNFP-VI</td><td>NeuAcα-6Galß-4GlcNAcß-3Galß-4Glc-DH
|
Fucα-3</td><td>2,840</td><td>11,119</td><td>476</td></tr>
<tr><td>82</td><td>GSC-97</td><td>NeuAcα-6Galß-4GlcNAcß-3Galß-4Glcß-Cer36
|
Fucα-3</td><td>342</td><td>1,759</td><td>70</td></tr>
<tr><td>83</td><td>MSLNH</td><td>NeuAcα-6Galß-4GlcNAcß-6
|
Galß-4Glc-DH
|
Galß-3GlcNAcß-3</td><td>4,703</td><td>9,262</td><td>254</td></tr>
</table>

**84**	**MSLNnH-I**	Galß-4GlcNAcß-6<br>| Galß-4Glc-DH<br>| NeuAcα-6Galß-3GlcNAcß-3	6,986	12,187	408
**85**	**DSLNnH**	NeuAcα-6Galß-4GlcNAcß-6<br>| Galß-4Glc-DH<br>| NeuAcα-6Galß-4GlcNAcß-3	8,271	14,070	430
**86**	**MFMSLNnH**	Galß-4GlcNAcß-6<br>| | Fucα-3 Galß-4Glc-DH<br>| NeuAcα-6Galß-3GlcNAcß-3	3,601	7,077	-
**87**	**A2(2-6)**	NeuAcα-6Galß-4GlcNAcß-2Manα-6<br>| Manß-4GlcNAcß-4GlcNAc-DH<br>| NeuAcα-6Galß-4GlcNAcß-2Manα-3	15,725	15,948	671
**88**	**BSM-Di-A1-AO**	NeuGcα-6GalNAc-AO	-	264	13,982
**89**	**BSM-Di-A2-AO**	NeuAcα-6GalNAc-AO	47	7,328	3,815
**2-3&2-6-linked sialyl**					
**90**	**DST**	NeuAcα-3Galß-3GalNAc-DH<br>| NeuAcα-6	372	5,792	2,802
**91**	**DST-AO**	NeuAcα-3Galß-3GalNAc-AO<br>| NeuAcα-6	493	10,142	8,591
**92**	**DSLNT**	NeuAcα-3Galß-3GlcNAcß-3Galß-4Glc-AO<br>| NeuAcα-6	6,953	16,602	7,733
**93**	**A3**	NeuAcα-3Galß-4GlcNAcß-2Manα-6<br>| Manß-4GlcNAcß-4GlcNAc-DH<br>| NeuAcα-3Galß-4GlcNAcß-4Manα-3<br>| NeuAcα-6Galß-4GlcNAcß-2	8,656	14,683	3,731
**2-8-linked sialyl**					
**94**	**GD3**	NeuAcα-8NeuAcα-3Galß-4Glcß-Cer	99	517	-
**95**	**GD3-tetra**	NeuAcα-8NeuAcα-3Galß-4Glc-DH	-	321	-
**96**	**GD3-tetra-AO**	NeuAcα-8NeuAcα-3Galß-4Glc-AO	-	234	-
**97**	**GD2**	GalNAcß-4Galß-4Glcß-Cer<br>| NeuAcα-8NeuAcα-3	-	165	-
**98**	**GD1b**	Galß-3GalNAcß-4Galß-4Glcß-Cer<br>| NeuAcα-8NeuAcα-3	-	217	-
**99**	**GQ1b**	NeuAcα-8NeuAcα-3Galβ-3GalNAcβ-4Galβ-4Glcβ-Cer<br>| NeuAcα-8NeuAcα-3	-	1,624	-
**100**	**SA2(α8)**	NeuAcα-8NeuAc-DH	-	210	-
**101**	**SA3(α8)**	NeuAcα-8NeuAcα-8NeuAc-DH	-	510	87
**102**	**SA4(α8)**	NeuAcα-8NeuAcα-8NeuAcα-8NeuAc-DH	-	467	1,296

**103**	**SA5(α8)**	NeuAcα-8NeuAcα-8NeuAcα-8NeuAcα-8NeuAc-DH***	11	516	1,823
**104**	**SA6(α8)**	NeuAcα-8NeuAcα-8NeuAcα-8NeuAcα-8NeuAcα-8NeuAc-DH***	27	502	817
**105**	**SA7(α8)**	NeuAcα-8NeuAcα-8NeuAcα-8NeuAcα-8NeuAcα-8NeuAcα-8NeuAc-DH***	-	447	717
**106**	**SA8(α8)**	NeuAcα-8NeuAcα-8NeuAcα-8NeuAcα-8NeuAcα-8NeuAc-8NeuAcα-8NeuAc-DH***	-	237	758
**107**	**SA9(α8)**	NeuAcα-8NeuAcα-8NeuAcα-8NeuAcα-8NeuAcα-8NeuAc-8NeuAcα-8NeuAc-8NeuAcα-DH***	-	416	845
**108**	**SA10(α8)**	NeuAcα-8NeuAcα-8NeuAcα-8NeuAcα-8NeuAcα-8NeuAc-8NeuAcα-8NeuAcα-8NeuAcα-8NeuAc-DH***	34	124	461
**2-3&2-8-linked sialyl**					
**109**	**GT1b**	NeuAcα-3Galβ-3GalNAcβ-4Galβ-4Glcβ-Cer<br>| NeuAcα-8NeuAcα-3	8,121	28,174	11,032
**110**	**GT1a**	NeuAcα-8NeuAcα-3Galβ-3GalNAcβ-4Galβ-4Glcβ-Cer<br>| NeuAcα-3	88	2,114	-
**2-3- or 2-6-linked sialyl**					
**111**	**SA(3/6)LNFP-I**	NeuAcα-3/6Galß-3GlcNAcß-3Galß-4Glc-DH<br>| Fucα-2	15	464	-
**112**	**AGP-Bi-Ac2**	NeuAcα-Galß-4GlcNAcß-2Manα-6<br>| Manß-4GlcNAcß-4GlcNAc-DH<br>| NeuAcα-Galß-4GlcNAcß-2Manα-3	10,231	14,328	1,836
**113**	**AGP-Bi-AcGc**	NeuGcα-Galß-4GlcNAcß-2Manα-6<br>? | Manß-4GlcNAcß-4GlcNAc-DH<br>| NeuAcα-Galß-4GlcNAcß-2Manα-3	2,543	6,912	1,387
**114**	**AGP-Bi-Gc2**	NeuGcα-Galß-4GlcNAcß-2Manα-6<br>| Manß-4GlcNAcß-4GlcNAc-DH<br>| NeuGcα-Galß-4GlcNAcß-2Manα-3	59	507	1,718
**2-9-linked sialyl**					
**115**	**GSC-96**	NeuAcα-9NeuAcα-3Galβ-4Glcß-Cer36	931	8,087	1,279

*The binding signals shown are the mean values at 5 fmol/spot with EtMIC3-MAR5 and TgMIC1-MARR.

**The oligosaccharide probes are lipid-linked, and are from the collection assembled in the course of research in Glycosciences Laboratory. DH, are NGLs prepared from reducing oligosaccharides by reductive amination (Chai et al., Methods Enzymol. 362, 160-195, 2003). AO, NGLs prepared from reducing oligosaccharides by oxime ligation with an aminooxy (AO) functionalized DHPE (Liu et al., Chem. Biol. 14, 847–859, 2007); Cer, natural glycolipids with various ceramide moieties; Cer36, synthetic glycolipids with ceramide having a total of 36 carbon atoms; C30, a synthetic lipid [2-(tetradecyl)hexadecanol] with 30 carbon atoms. NB Neu denotes de-N-acetylated neuraminic acid.

*** Major component.

C30, DH, AO, Cer, and Cer36 refer to different lipid tags of glycan probes. These are defined in Supplemental Table
