# Supplementary material for: The Role of Sialyl Glycan Recognition in Host Tissue Tropism of the Avian Parasite Eimeria tenella
Source: PLoS Pathog. 2011 Oct 13;7(10):e1002296. doi: 10.1371/journal.ppat.1002296 (PMC3192848; doi:10.1371/journal.ppat.1002296)
Supplement: Table S2 — Intermolecular NOE restraints for HADDOCK docking calculations of EtMIC3 with sialyl carbohydrates. (PDF) [file ppat.1002296.s007.pdf]

**Intermolecular NOE restraints for HADDOCK docking calculations of EtMIC3 with sialyl carbohydrates**

| NOE | Protein atom(s) | Carbohydrate atom(s) |
|---|---|---|
| **1** | His 86 $\delta_2$H | Sia H* |
| **2** | Leu 24 $\delta CH_3$ | Sia HC4* |
| **3** | Leu 24 $\delta CH_3$ | Sia HN4 |
| **4** | Leu 24 $\delta CH_3$ | Sia H2, H4, H5, H6, H7 or H8 |
| **5** | Leu 87 $\delta CH_3$ | Sia HN4 |
| **6** | Leu 87 $\delta CH_3$ | SIA H2, H4, H5, H6, H7 or H8 |
| **7** | Thr 88 $\gamma 2CH_3$ | SIA H2, H4, H5, H6, H7 or H8 |
| **8** | Thr 84 $\gamma 2CH_3$ | Sia HC4* |
| **9** | Thr 84 $\gamma 2CH_3$ | Sia HN4 |
