# Supplementary material for: The Role of Sialyl Glycan Recognition in Host Tissue Tropism of the Avian Parasite Eimeria tenella
Source: PLoS Pathog. 2011 Oct 13;7(10):e1002296. doi: 10.1371/journal.ppat.1002296 (PMC3192848; doi:10.1371/journal.ppat.1002296)
Supplement: Table S3 — Cumulative faecal oocysts counts from each individual bird in the two vaccination/challenge experiments shown in Figure 7 . (PDF) [file ppat.1002296.s008.pdf]

Cumulative faecal oocysts counts from each individual bird in the two vaccination/challenge experiments shown in Figure 7.

Experiment a) Protein vaccination

Cumulative faecal oocyst counts days 6-11 post challenge			
**Vaccination treatment**	**Total oocyst ouput per bird**	**Average**	**SEM**
**EtMIC3-MAR1c**	2.56E+07		
**EtMIC3-MAR1c**	1.49E+07		
**EtMIC3-MAR1c**	8.58E+06		
**EtMIC3-MAR1c**	1.17E+07		
**EtMIC3-MAR1c**	6.80E+06	1.35E+07[a]	3.32E+06
**Thioredoxin**	2.27E+07		
**Thioredoxin**	4.15E+07		
**Thioredoxin**	2.88E+07		
**Thioredoxin**	2.42E+07		
**Thioredoxin**	3.04E+07	2.95E+07[b]	3.31E+06
**PBS**	1.98E+07		
**PBS**	3.97E+07		
**PBS**	4.40E+07		
**PBS**	6.80E+07		
**PBS**	2.11E+07	3.85E+07[b]	8.82E+06

[a, b,] Averages annotated with superscript letter ‘a’ are not significantly different from each other, but are significantly different from those labeled with ‘b’ ($p<0.05$, Oneway ANOVA and post -hoc Tukey test), and vice versa.

Experiment b) DNA vaccination

Cumulative faecal oocysts counts days 5-9 post challenge			
**Vaccination treatment**	**Total oocyst output per bird**	**Average**	**SEM**
**MIC3MAR5**	1.18E+07		
**MIC3MAR5**	5.86E+06		
**MIC3MAR5**	2.30E+06		
**MIC3MAR5**	1.36E+06		
**MIC3MAR5**	1.39E+07		
**MIC3MAR5**	2.12E+07	9.40E+06[a]	3.12E+06
**MIC3MAR1**	7.66E+06		
**MIC3MAR1**	1.42E+07		
**MIC3MAR1**	7.44E+06		
**MIC3MAR1**	8.48E+06		
**MIC3MAR1**	9.28E+06		
**MIC3MAR1**	6.00E+06	8.84E+06[a]	1.16E+06
**pcDNA**	4.13E+07		
**pcDNA**	1.04E+07		
**pcDNA**	1.98E+07		
**pcDNA**	5.20E+06		
**pcDNA**	9.82E+06		
**pcDNA**	2.24E+07	1.82E+07[b]	5.33E+06
**PBS**	8.28E+06		
**PBS**	1.95E+07		
**PBS**	1.10E+07		
**PBS**	1.73E+07		
**PBS**	1.66E+07		
**PBS**	2.12E+07	1.56E+07[b]	2.04E+06

[a, b,] Averages annotated with superscript letter ‘a’ are not significantly different from each other, but are significantly different from those labeled with ‘b’ ($p<0.05$, Oneway ANOVA and post -hoc Tukey test), and vice versa.
